# Cardioprotective effect of combination therapy by mild hypothermia and local or remote ischemic preconditioning in isolated rat hearts

**DOI:** 10.1038/s41598-020-79449-x

**Published:** 2021-01-11

**Authors:** Marie V. Hjortbak, Nichlas R. Jespersen, Rebekka V. Jensen, Thomas R. Lassen, Johanne Hjort, Jonas A. Povlsen, Nicolaj B. Støttrup, Jakob Hansen, Derek J. Hausenloy, Hans Erik Bøtker

**Affiliations:** 1 Department of Cardiology, Aarhus University Hospital, Palle Juul-Jensens Boulevard 99, Aarhus N, Denmark; 2 Department of Forensic Medicine, Aarhus University Hospital, Aarhus, Denmark; 3 Cardiovascular & Metabolic Disorders Program, Duke-National University of Singapore Medical School, Singapore, Singapore; 4 National Heart Research Institute Singapore, National Heart Centre, Singapore, Singapore; 5 Yong Loo Lin School of Medicine, National University Singapore, Singapore, Singapore; 6 The Hatter Cardiovascular Institute, University College London, London, UK; 7 Cardiovascular Research Center, College of Medical and Health Sciences, Asia University, Taichung City, Taiwan

**Keywords:** Cardiology, Medical research

## Abstract

A multitargeted strategy to treat the consequences of ischemia and reperfusion (IR) injury in acute myocardial infarction may add cardioprotection beyond reperfusion therapy alone. We investigated the cardioprotective effect of mild hypothermia combined with local ischemic preconditioning (IPC) or remote ischemic conditioning (RIC) on IR injury in isolated rat hearts. Moreover, we aimed to define the optimum timing of initiating hypothermia and evaluate underlying cardioprotective mechanisms. Compared to infarct size in normothermic controls (56 ± 4%), mild hypothermia during the entire or final 20 min of the ischemic period reduced infarct size (34 ± 2%, p < 0.01; 35 ± 5%, p < 0.01, respectively), while no reduction was seen when hypothermia was initiated at reperfusion (51 ± 4%, p = 0.90). In all groups with effect of mild hypothermia, IPC further reduced infarct size. In contrast, we found no additive effect on infarct size between hypothermic controls (20 ± 3%) and the combination of mild hypothermia and RIC (33 ± 4%, p = 0.09). Differences in temporal lactate dehydrogenase release patterns suggested an anti-ischemic effect by mild hypothermia, while IPC and RIC preferentially targeted reperfusion injury. In conclusion, additive underlying mechanisms seem to provide an additive effect of mild hypothermia and IPC, whereas the more clinically applicable RIC does not add cardioprotection beyond mild hypothermia.

## Introduction

Major advances in the treatment of acute myocardial infarction (MI) have reduced immediate mortality rates^1^. Among the growing number of patients surviving the acute phase of acute MI, post-MI congestive heart failure remains a significant cause of morbidity and mortality.

Myocardial infarct size is a main predictor of survival and clinical outcome in patients with acute MI. While the key treatment of MI is rapid reperfusion by primary percutaneous coronary intervention (PCI)^2^, reperfusion per se may itself cause myocardial injury by the paradoxical phenomenon known as reperfusion injury^3^. Hence, both ischemia and reperfusion injury determine final myocardial infarct size. Targeting ischemia/reperfusion (IR) injury beyond rapid revascularisation appears to be an important step towards improving outcomes in patients with acute MI. Pharmacological and mechanical conditioning and mild therapeutic hypothermia have been applied to attenuate IR-injury, but none of the approaches have individually translated into convincing clinical efficacy and reduced mortality^4^. A multitargeted approach may therefore be more effective as a cardioprotective strategy^5^.

Two cardioprotective modalities that have been shown to reduce infarct size as an adjunct to primary PCI include remote ischemic conditioning (RIC)^6^ and mild hypothermia^7–10^, but as distinct procedures the translation of these modalities into clinical efficacy also seems challenging^11,12^. A multitargeted approach by combining these clinically applicable methods is attractive in patients undergoing primary PCI, not only because the combination of methods may have an additive cardioprotective effect^13,14^, but also because patients with cardiac arrest due to acute MI are exposed to universal hypothermia of 33–36 °C prior to or following primary PCI. Hence, the optimal timing and underlying mechanisms of ischemic conditioning and mild hypothermia need to be defined.

The aims of the present study were to investigate whether mechanical conditioning by ischemic preconditioning (IPC) yield additive cardioprotection during mild hypothermia, to define the optimum timing for hypothermia, and identify underlying cardioprotective mechanisms. Finally, we elaborated the study with a separate series aiming to investigate a treatment strategy of RIC and mild hypothermia, which is a clinically more feasible add-on treatment to primary PCI.

## Results

### Experimental protocols

**Figure 1:**
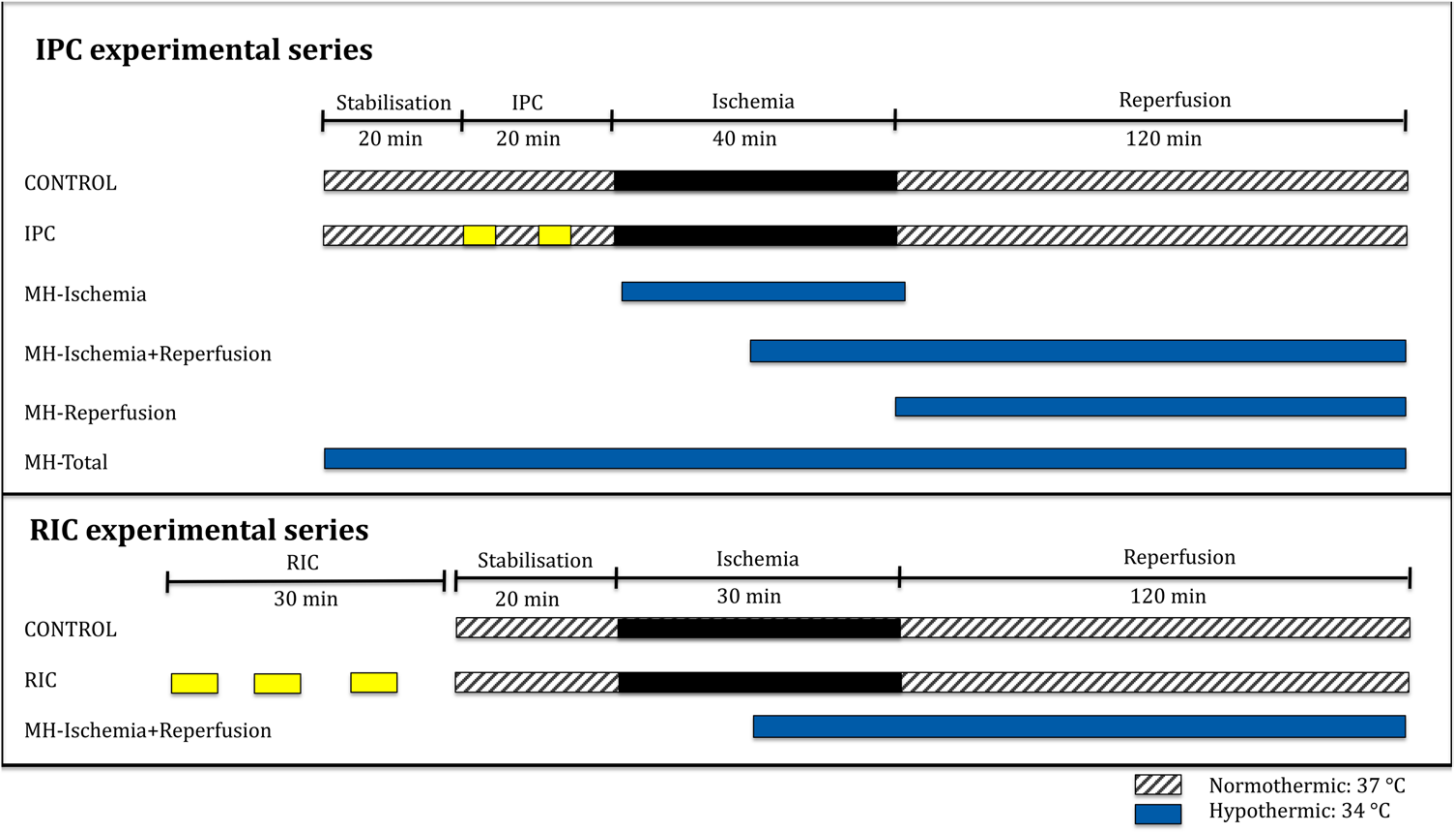
Study design. *CONTROL* control, *IPC* ischemic preconditioning, *RIC* remote ischemic preconditioning, *MH-Ischemia* mild hypothermia during ischemia, *MH-Ischemia + Reperfusion* mild hypothermia during last 20 min of ischemia and throughout reperfusion, *MH-Reperfusion* mild hypothermia during reperfusion only, *MH-Total* mild hypothermia through the total protocol.

The study was designed with two separate experimental series. First, an IPC experimental series investigated the effect of IPC in combination with mild hypothermia, and the timing of mild hypothermia. Second, a RIC experimental series investigated the combination of RIC and mild hypothermia (Fig. 1). All hearts were isolated and subjected to global ischemia and reperfusion in an isolated perfused heart model.

### Infarct size

**Figure 2:**
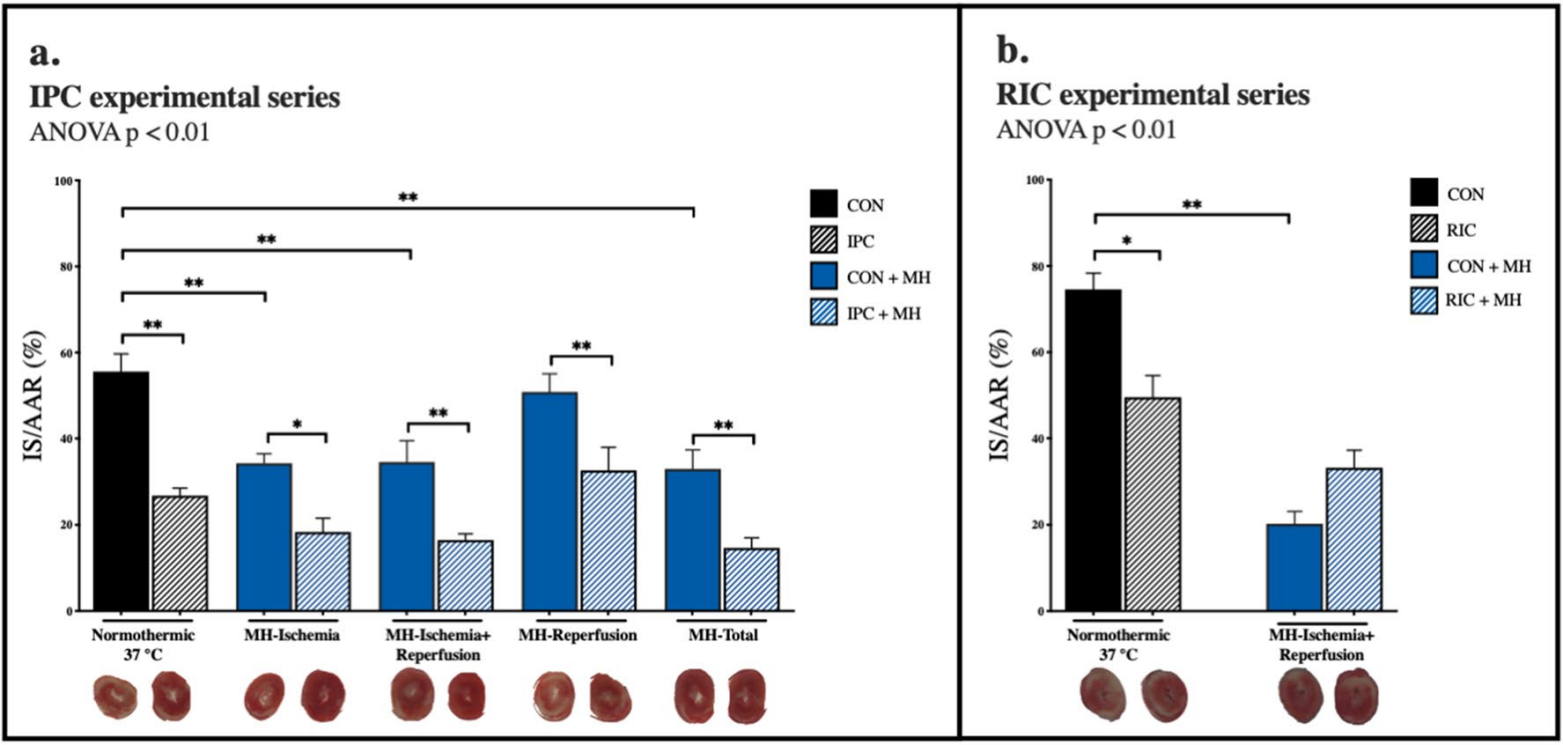
Infarct size. (**a**) IPC experimental series, (**b**) RIC experimental series. *IS/AAR* Infarct size/area at risk, *CON* control, *IPC* ischemic preconditioning, *RIC* remote ischemic preconditioning, *MH-Ischemia* mild hypothermia during ischemia, *MH-Ischemia + Reperfusion* mild hypothermia during last 20 min of ischemia and throughout reperfusion, *MH-Reperfusion* mild hypothermia during reperfusion, *MH-Total* mild hypothermia through the total protocol. *p < 0.05, **p < 0.01.

In the IPC experimental series infarct size was 56 ± 4% of the area-at-risk in the control group. Hypothermia applied during ischemia reduced infarct size independent of hypothermia duration, i.e. during the entire ischemic phase (34 ± 2%, p < 0.01), during the final 20 min of the ischemic phase plus reperfusion (35 ± 5%, p < 0.01), or during the total experimental period including stabilization, ischemia and reperfusion (33 ± 4%, p < 0.01) (Fig. 2a). In contrast, there was no infarct size reduction, when hypothermia was applied during reperfusion alone (51 ± 4%, p > 0.90) (Fig. 2a).

IPC reduced infarct size during normothermia (27 ± 2%, p < 0.01). IPC added further infarct size reduction beyond hypothermia, regardless of the timing of hypothermia (Fig. 2a) (MH-Ischemia: 18 ± 3%, p = 0.02; MH-Ischemia + Reperfusion: 16 ± 1%, p < 0.01; MH-Reperfusion: 33 ± 5%, p < 0.01 and MH-Total: 15 ± 2%, p < 0.01).

In the RIC experimental series infarct size was 75 ± 4% in the control group (Fig. 2b). RIC reduced infarct size during normothermia (50 ± 5%, p < 0.01), but not when given in combination with hypothermia during the final 20 min of ischemia and reperfusion (33 ± 4% vs. 20 ± 3% with hypothermia alone, p = 0.09).

### Cardiac function

#### Baseline

Hemodynamic variables are presented in Table 1a,b. In the IPC experimental series, normothermic IPC treated animals had significantly higher heart rate (HR) and rate pressure product (RPP) than normothermic controls at baseline. We found no other differences in HR, LVDP, or RPP between the experimental groups at baseline (Table 1a).

**Table 1:** Cardiac function.

	Baseline				Ischemia	Reperfusion				
	N	20 min	p-values			10 min	30 min	120 min	p-values	
			Control vs. intervention	Control-MH vs IPC-MH					Control vs. intervention	Control-MH vs IPC-MH
**a. IPC experimental series**										
**LVDP**			**ANOVA: 0.02**						**ANOVA: < 0.01**	
CON	7	151 ± 7				2 ± 1	17 ± 6	25 ± 4		
IPC	8	145 ± 4		0.99		5 ± 1	31 ± 5	35 ± 3		0.04
CON MH-ischemia	8	145 ± 4	0.80			10 ± 2	35 ± 7	33 ± 3	0.06	
IPC MH-ischemia	8	148 ± 3		0.97		35 ± 4	83 ± 4	48 ± 3		< 0.01
CON MH ischemia + reperfusion	8	148 ± 7	> 0.99			5 ± 1	27 ± 7	45 ± 5	0.06	
IPC MH-ischemia + reperfusion	8	154 ± 4		0.97		15 ± 4	72 ± 6	65 ± 3		< 0.01
CON MH-reperfusion	7	159 ± 6	0.94			1 ± 0	11 ± 3	34 ± 4	0.84	
IPC MH-reperfusion	8	158 ± 2		> 0.99		6 ± 3	52 ± 7	51 ± 5		0.02
CON MH-total	7	157 ± 4	0.98			6 ± 2	50 ± 9	49 ± 5	0.04	
IPC MH-total	8	165 ± 5		0.94		27 ± 7	92 ± 4	71 ± 2		< 0.01
**Heart rate (BPM)**			**ANOVA: < 0.01**						**ANOVA: < 0.01**	
CON	7	196 ± 22				199 ± 18	226 ± 24	239 ± 20		
IPC	8	264 ± 15		0.02		238 ± 11	227 ± 19	244 ± 12		0.46
CON MH-ischemia	8	235 ± 17	0.49			178 ± 19	227 ± 18	224 ± 14	0.61	
IPC MH-ischemia	8	210 ± 13		0.90		229 ± 22	219 ± 18	217 ± 15		0.55
CON MH ischemia + reperfusion	8	237 ± 17	0.45			215 ± 16	202 ± 14	174 ± 11	0.21	
IPC MH-ischemia + reperfusion	8	234 ± 10		> 0.99		244 ± 10	208 ± 10	202 ± 4		0.04
CON MH-reperfusion	7	196 ± 12	> 0.99			231 ± 27	186 ± 25	176 ± 19	0.33	
IPC MH-reperfusion	8	192 ± 14		> 0.99		223 ± 29	156 ± 19	160 ± 13		0.41
CON MH-total	7	175 ± 15	0.98			191 ± 15	163 ± 15	183 ± 10	0.06	
IPC MH-total	8	156 ± 11				175 ± 22	165 ± 13	184 ± 13		0.80
**Rate pressure product (BPM×mmHg)**			**ANOVA: < 0.01**						**ANOVA: < 0.01**	
CON	7	29,127 ± 2586				506 ± 117	3738 ± 1307	5928 ± 1064		
IPC	8	38,004 ± 1594		0.05		1267 ± 279	6946 ± 1305	8559 ± 905		0.06
CON MH-ischemia	8	33,843 ± 2417	0.72			1611 ± 453	7786 ± 1684	7304 ± 917	0.10	
IPC MH-ischemia	8	31,019 ± 1926		0.98		7708 ± 881	17,977 ± 1605	10,137 ± 633		< 0.01
CON MH ischemia + reperfusion	8	35,213 ± 3125	0.39			972 ± 146	5103 ± 1292	7980 ± 1162	0.26	
IPC MH-ischemia + reperfusion	8	35,976 ± 1578		> 0.99		3620 ± 787	14,621 ± 1117	13,209 ± 627		< 0.01
CON MH-reperfusion	7	30,960 ± 1434	> 0.99			295 ± 77	1863 ± 534	5918 ± 738	0.43	
IPC MH-reperfusion	8	30,345 ± 2242		> 0.99		1403 ± 810	5730 ± 1147	7954 ± 856		0.03
CON MH-total	7	27,483 ± 2131	> 0.99			1214 ± 397	5318 ± 1636	8848 ± 991	0.19	
IPC MH-total	8	25,587 ± 1643		> 0.99		3673 ± 534	15,301 ± 1428	12,969 ± 951		< 0.01
**Coronary flow**			**ANOVA: < 0.01**						**ANOVA: 0.01**	
CON	7	11 ± 0				11 ± 1	10 ± 0	9 ± 0		
IPC	8	14 ± 1		0.23		15 ± 1	13 ± 1	12 ± 1		0.04
CON MH-ischemia	8	11 ± 1	> 0.99			10 ± 1	9 ± 1	9 ± 1	0.11	
IPC MH-ischemia	8	10 ± 1		> 0.99		13 ± 1	11 ± 1	8 ± 1		< 0.01
CON MH ischemia + reperfusion	8	12 ± 1	0.96			13 ± 1	12 ± 1	8 ± 1	0.18	
IPC MH-ischemia + reperfusion	8	12 ± 1		> 0.99		13 ± 1	11 ± 1	8 ± 1		< 0.01
CON MH-reperfusion	7	12 ± 1	0.99			10 ± 1	10 ± 1	10 ± 1	0.45	
IPC MH-reperfusion	8	10 ± 1		0.48		12 ± 1	10 ± 1	11 ± 1		< 0.01
CON MH-total	7	10 ± 1	0.82			10 ± 1	9 ± 1	8 ± 1	0.16	
IPC MH-total	8	9 ± 1		> 0.99		± 1	10 ± 0	9 ± 0		< 0.01
**b. RIC experimental series**										
**LVDP**			**ANOVA: 0.09**						**ANOVA: < 0.01**	
CON	7	145 ± 6				4 ± 1	18 ± 5	15 ± 1		
RIC	8	121 ± 20				7 ± 2	34 ± 2	25 ± 2		0.02
CON MH-ischemia + reperfusion	8	158 ± 3				14 ± 4	62 ± 4	52 ± 5	< 0.01	
RIC MH-ischemia + reperfusion	8	155 ± 3				14 ± 4	51 ± 5	41 ± 4		0.2
**Heart rate (BPM)**			**ANOVA: 0.98**						**ANOVA: < 0.01**	
CON	7	244 ± 13				224 ± 29	251 ± 16	259 ± 10		
RIC	8	246 ± 18				235 ± 15	263 ± 11	259 ± 11		0.39
CON MH-Ischemia + Reperfusion	8	252 ± 15				179 ± 27	176 ± 12	180 ± 15	0.02	
RIC MH-Ischemia + Reperfusion	8	242 ± 24				174 ± 24	196 ± 17	202 ± 12		0.75
**Rate pressure product (BPM × mmHg)**			**ANOVA: 0.23**						**ANOVA: < 0.01**	
CON	7	35,231 ± 2282				747 ± 217	5516 ± 724	3825 ± 365		
RIC	8	29,450 ± 5257				1768 ± 576	8923 ± 448	6495 ± 611		0.01
CON MH-ischemia + reperfusion	8	39,871 ± 2693				2577 ± 895	10,599 ± 896	9229 ± 1182	< 0.01	
RIC MH-ischemia + reperfusion	8	37,124 ± 3219				1930 ± 525	9792 ± 877	8143 ± 831		0.15
**Coronary flow**			**ANOVA: 0.14**						**ANOVA: 0.25**	
CON	7	13 ± 1				10 ± 1	8 ± 1	7 ± 1		
RIC	8	11 ± 1				9 ± 1	7 ± 1	6 ± 1		
CON MH-ischemia + reperfusion	8	14 ± 1				12 ± 1	9 ± 1	7 ± 1		

(a) IPC experimental series, (b) RIC experimental series.

*CON* control, *IPC* ischemic preconditioning, *RIC* remote ischemic preconditioning, *MH-Ischemia* mild hypothermia during ischemia, *MH-Ischemia + Reperfusion* mild hypothermia during half of the ischemia and throughout reperfusion, *MH-Reperfusion* mild hypothermia during reperfusion, *MH-Total* mild hypothermia through the total protocol.

In the RIC experimental series (Table 1b), we found no hemodynamic differences between any of the experimental groups of the combination therapy of RIC and mild hypothermia at baseline.

#### Reperfusion

Hypothermia during the entire protocol improved hemodynamic recovery with increased LVDP compared to normothermic controls. Hypothermia during ischemia alone or final 20 min of ischemia including reperfusion also increased LVDP, but not statistically significantly. HR and RPP were lowered slightly by mild hypothermia, but not statistically significantly. Hypothermia had no effect on LVDP or RPP, when hypothermia was given only during reperfusion.

IPC improved hemodynamic recovery significantly in normothermic animals by increasing LVDP during reperfusion, and IPC had additive effect with increased LVDP in hypothermic animals. While IPC did not change RPP significantly in normothermic animals, IPC increased RPP in all cooling protocols compared to their respective hypothermic control groups.

In the RIC experimental series, mild hypothermia increased LVDP and RPP compared to normothermia.

RIC also improved hemodynamic recovery in normothermic animals with increased LVDP and RPP but not during mild hypothermia (Table 1b).

### Biochemical markers of myocardial ischemia (temporal LDH release)

**Figure 3:**
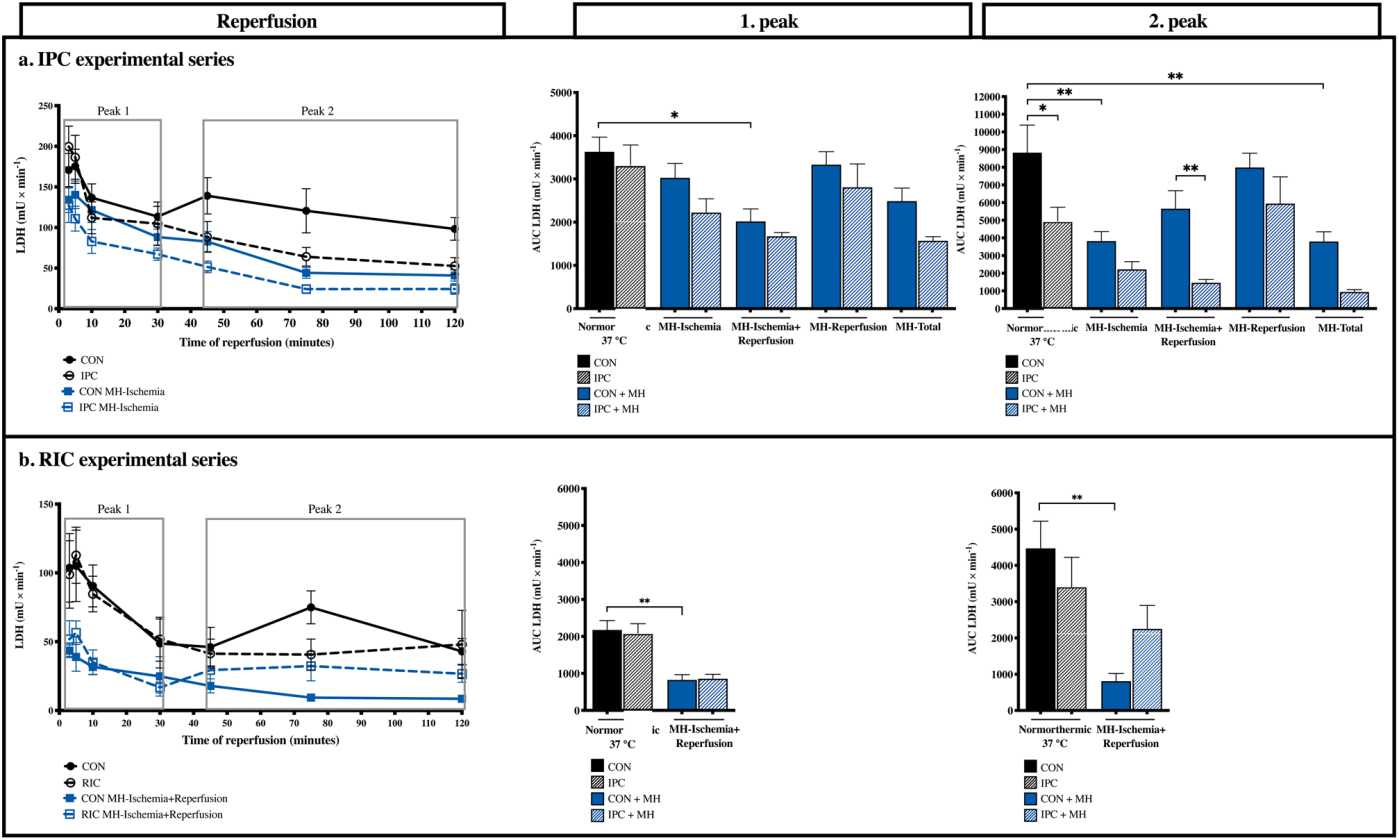
LDH release. (**a**) IPC experimental series, (**b**) RIC experimental series. *LDH* Lactate dehydrogenase, *AUC* area under the curve, *CON* control, *IPC* ischemic preconditioning, *RIC* remote ischemic preconditioning, *MH-Ischemia* mild hypothermia during ischemia, *MH-Ischemia + Reperfusion* mild hypothermia during last 20 min of ischemia and throughout reperfusion, *MH-Reperfusion* mild hypothermia during reperfusion, *MH-Total* mild hypothermia through the total protocol. *p < 0.05, **p  < 0.01.

Continuous sampling of effluent during reperfusion showed that lactate dehydrogenase (LDH) was released in a biphasic pattern with two distinct peaks (Fig. 3). The initial peak was observed during the first 30 min of reperfusion, and included samples from 3, 5, 10, and 30 min of reperfusion. The second peak was observed during last part of the reperfusion, and included sample points from 45, 75, and 120 min of reperfusion.

Mild hypothermia during the last 20 min of ischemia and the entire reperfusion reduced the first peak in LDH release compared to normothermic controls (p = 0.01), while this was not the case for mild hypothermia during either ischemia, reperfusion, or during the total protocol (p = 0.9, p > 0.9 and p = 0.2, respectively) (Fig. 3a). Although addition of IPC resulted in slightly lower first peaks during normo- and hypothermia, neither reduction was statistically significant (Normorthermia: p = 0.9, MH-Ischemia: p = 0.6, MH-Ischemia + Reperfusion: p > 0.9, MH-Reperfusiom: p > 0.9 and MH-Total: p = 0.4). RIC did not change the magnitude of the first peak (p > 0.9) (Fig. 3b).

IPC reduced the second peak during normothermia (p = 0.02), and during all hypothermic protocols applied during ischemia, although only with statistical significance in the group with hypothermia during the last 20 min of ischemia plus reperfusion (p < 0.01) (Fig. 3a). RIC did not reduce the second peak during normothermia significantly (p = 0.60) (Fig. 3b).

### Microdialysis

#### Lactate

**Figure 4:**
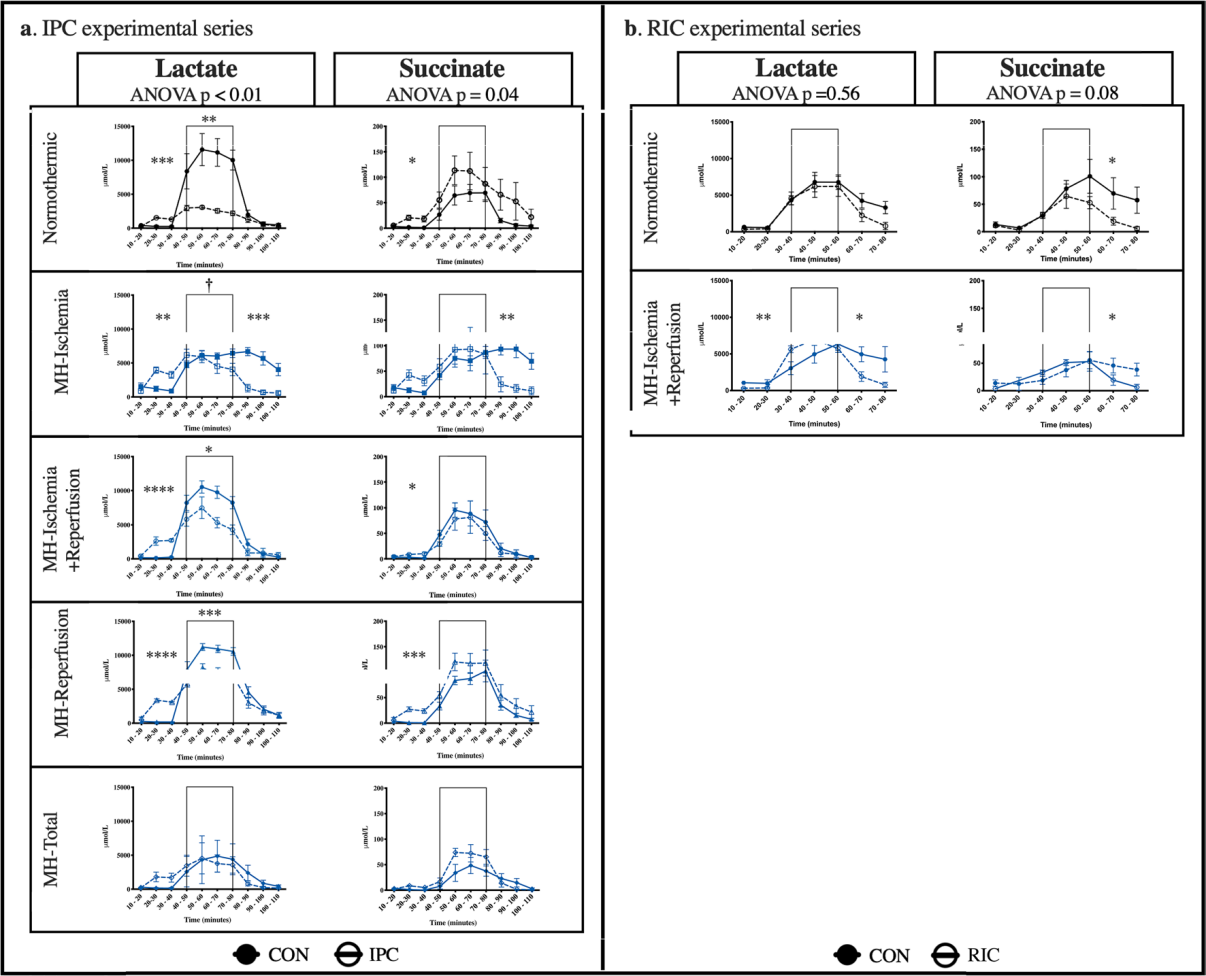
Microdialysis. (**a**) IPC experimental series, (**b**) RIC experimental series. *CON* control, *IPC* ischemic preconditioning, *RIC* remote ischemic preconditioning, *MH-Ischemia* mild hypothermia during ischemia, *MH-Ischemia + Reperfusion* mild hypothermia for last 20 min of ischemia and throughout reperfusion, *MH-Reperfusion* mild hypothermia during reperfusion, *MH-Total* mild hypothermia through the total protocol. ^†^P represents comparison between normothermic CON and CON MH-groups. *p represents comparison of CON and IPC according to MH-protocol. *p < 0.05, **p < 0.01, ***p < 0.001, ****p < 0.0001.

Mild hypothermia attenuated the increase in lactate release during ischemia when mild hypothermia was applied during the whole ischemic period (p = 0.03) and the total experiment (p = 0.09) (Fig. 4a). Mild hypothermia had no impact on lactate release when given during the last 20 min of ischemia plus reperfusion or during reperfusion only.

IPC reduced lactate release during ischemia under normothermic conditions (p < 0.01). Lactate release during ischemia was reduced by IPC in the MH-Reperfusion group (p = 0.02). In MH-Total group, IPC had no additional effect on the lactate release, which was pronouncedly suppressed during the entire experiment.

During normothermia RIC tended to enhance the decrement during reperfusion (p = 0.07), but RIC did not affect lactate release during ischemia (p = 0.83) (Fig. 4b). In combination with mild hypothermia, RIC did not change lactate release during ischemia (p = 0.15) but further reduced lactate release compared to hypothermia alone during reperfusion (p = 0.04).

#### Succinate

Succinate release increased during global ischemia with a subsequent decrease during reperfusion (Fig. 4). None of the mild hypothermia protocols changed succinate release during ischemia, but the succinate release was extended to the reperfusion period in CON MH-Ischemia group (Fig. 4a). IPC abrogate this extension (p < 0.01). IPC did not affect succinate release during ischemia or reperfusion, even though succinate was statistically non-significantly higher during the last part of ischemia in the normothermic group compared to the IPC group.

RIC accelerated the decrement in succinate release reperfusion during normothermia (p = 0.04). RIC in combination with hypothermia reduced succinate levels to the same extent as hypothermia alone (p = 0.03) (Fig. 4b).

### Glucose oxidation

**Figure 5:**
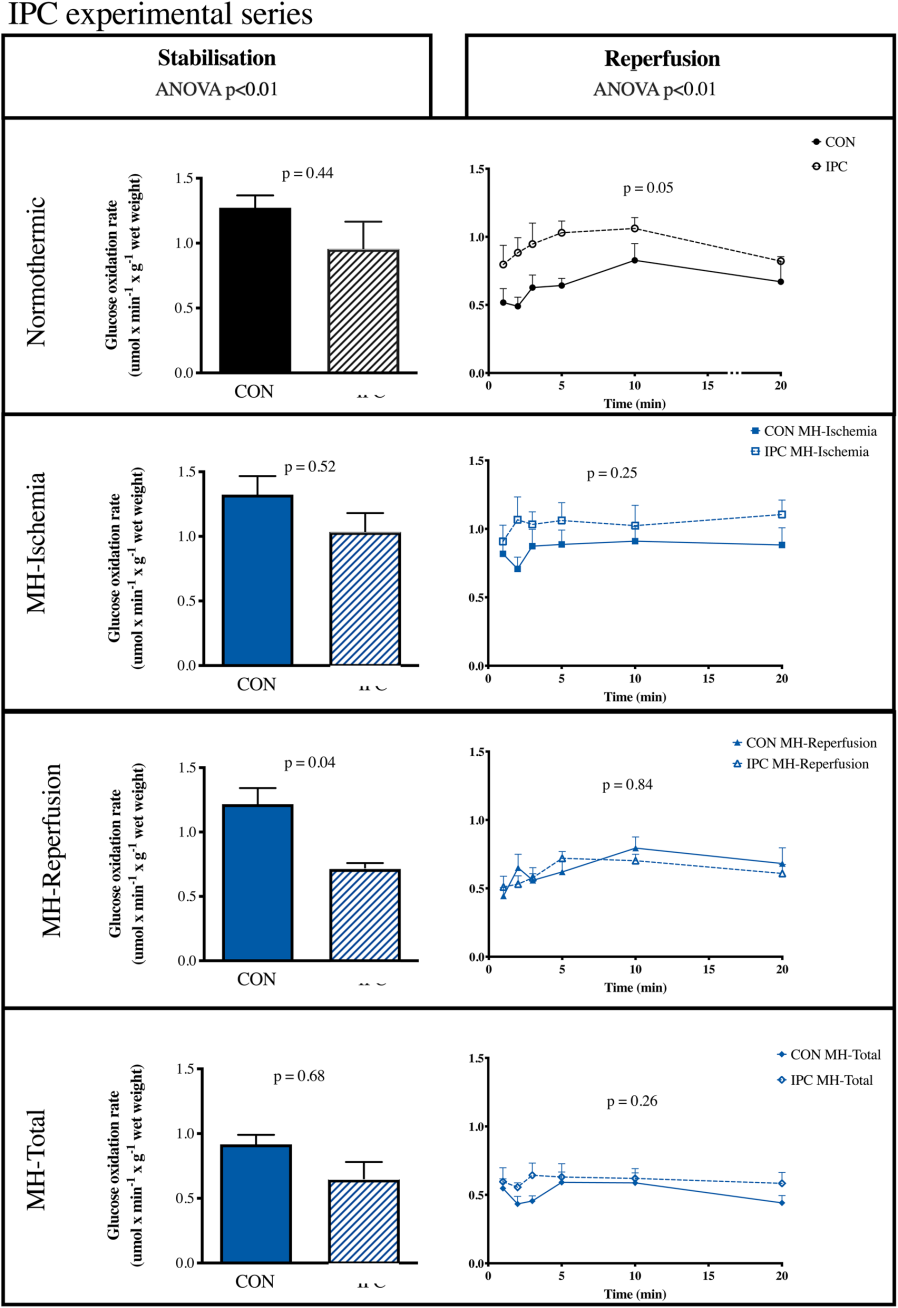
Glucose oxidation. *CON* control, *IPC* ischemic preconditioning, *MH-Ischemia* mild hypothermia during ischemia, *MH-Reperfusion* mild hypothermia during reperfusion, *MH-Total* mild hypothermia through the total protocol.

Pre-ischemic glucose oxidation was slightly lower after IPC than in controls, but only statistically significantly in the MH-Reperfusion group (Fig. 5). In the RIC series, glucose oxidation during stabilisation was similar in all groups (see Supplementary Information).

Compared to normothermia, mild hypothermia modified post-ischemic glucose oxidation only when applied during ischemia alone (p < 0.01) (Fig. 5). IPC increased glucose oxidation during reperfusion in the normothermic group (p = 0.05) (Fig. 5), but not when combined with mild hypothermia.

RIC did not change glucose oxidation during reperfusion (p = 0.30) (Supplementary Fig. S1).

### Intracellular signalling pathways

**Figure 6:**
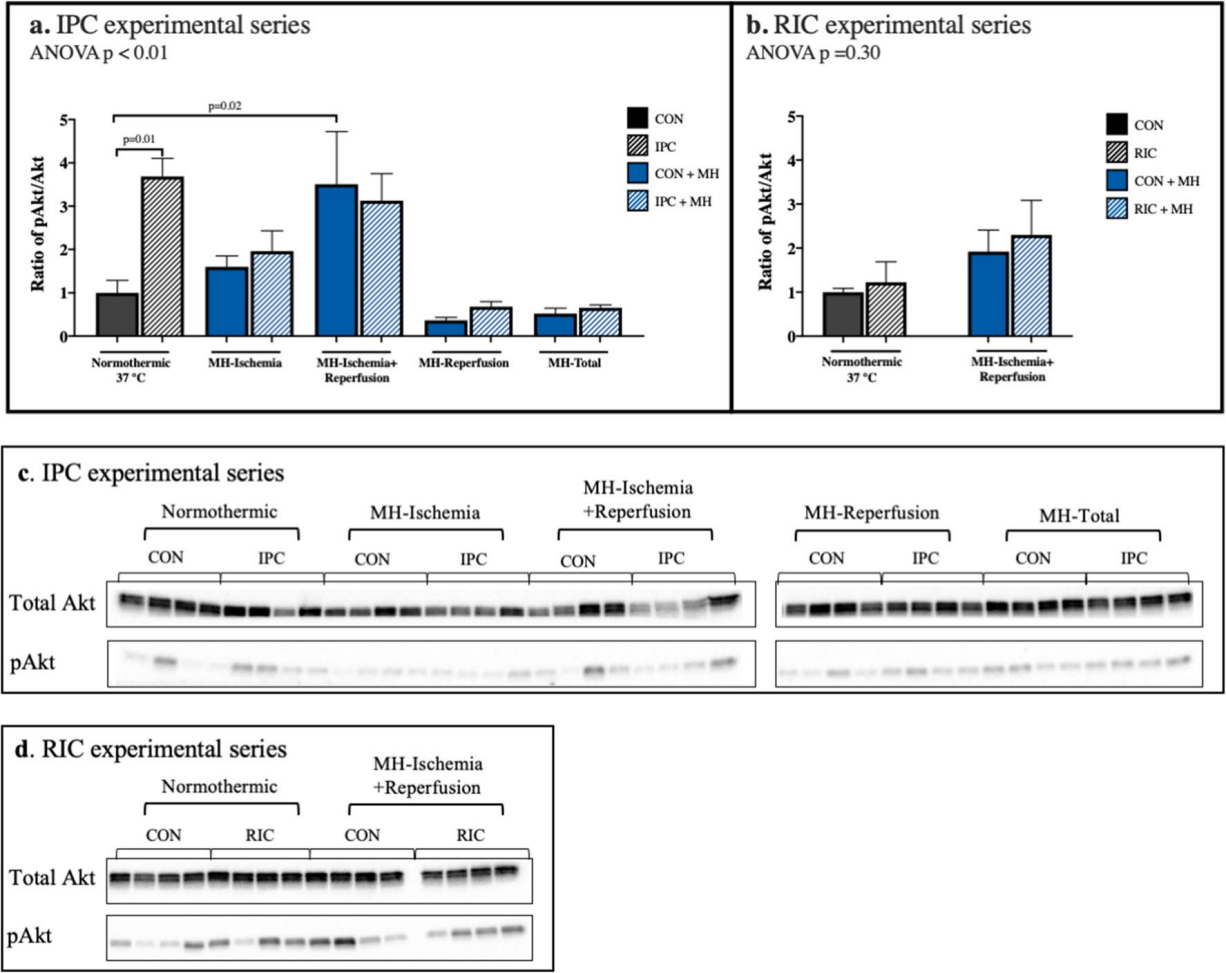
Phosphorylation of Akt. The ratio of pAkt/total Akt (60 kDa). (**a**) IPC experimental series, (**b**) RIC experimental series, (**c**) representative blots from IPC experimental series, data represents two separate blots. (**d**) Representative blots from RIC experimental series. *CON* control, *IPC* ischemic preconditioning, *RIC* remote ischemic preconditioning, *MH-Ischemia* mild hypothermia during ischemia, *MH-Ischemia + Reperfusion* mild hypothermia for last 20 min of ischemia and throughout reperfusion, *MH-Reperfusion* mild hypothermia during reperfusion, *MH-Total* mild hypothermia through the total protocol. Data are presented as a ratio with normothermic CON as reference group. N = 4 in all groups.

Compared to normothermia, mild hypothermia increased the pAkt/Akt ratio more than threefold (p = 0.02) when applied during the final 20 min of ischemia and throughout reperfusion (Fig. 6a). IPC increased pAkt/Akt ratio almost fourfold during normothermia compared to controls (p = 0.01) but had no additive effect to MH (Fig. 6a).

In the RIC experimental series, no significant differences in phosphorylation of Akt was found (ANOVA p = 0.30) (Fig. 6b).

Representative blots for both experimental series are shown in Fig. 6c,d.

We detected no statistically significant differences in phosphorylation of ERK by hypothermia, IPC or RIC (Supplementary Fig. S2).

## Discussion

The main findings of the present study are that IPC had a preserved and even additive cardioprotective effect when combined with mild hypothermia during ischemia, while RIC did not. If these findings can be translated to the clinical scenario, our findings indicate that IPC may yield additional cardioprotection during e.g. cardiac surgery with hypothermic cardioplegia. A multitargeted strategy including RIC and mild hypothermia does not seem to yield additional cardioprotective effect beyond either intervention alone.

We extend previous findings that the cardioprotective effect of hypothermia is only operative when applied during ischemia^15–20^, and not when applied during reperfusion^16,19,21^ by demonstrating that hypothermia is efficient, when applied during a final period of the ischemic event. While in our setting this period was 20 min, Götberg et al. demonstrated that protection was achieved by mild hypothermia during only the final 5 min of ischemia in a model of LAD occlusion in pigs^22^.

The hemodynamic measurements reflected the final infarct size as hemodynamic performance improved with reduced infarct size. At baseline, the hearts performed similarly in the corresponding control and intervention groups. However, normothermic IPC-treated hearts showed a higher baseline HR than their normothermic controls. We did not observe any similar response in the other IPC groups and believe that our finding is incidental.

We used frequent analyses of LDH release during reperfusion to clarify whether the cardioprotective effect of mild hypothermia was mainly associated with the ischemic-injury or the reperfusion-injury. We have previously shown that LDH is released in two distinct peaks during reperfusion in the isolated heart model^23^. The first peak is associated with ischemic-injury, while the second peak is associated mainly with reperfusion-injury. Mild hypothermia involving the ischemic period generally reduced the release of LDH during reperfusion, but the peaks were affected differently depending on the protocol: when the whole ischemic period was hypothermic followed by normothermic reperfusion, the second peak was reduced, while hypothermia during only the final 20 min of the ischemic period followed by hypothermic reperfusion reduced the first peak. Moreover, hypothermia during reperfusion alone did not affect LDH release during reperfusion. We interpret these results to demonstrate that mild hypothermia has a cardioprotective effect during ischemia and that ischemic and reperfusion-injury are tightly connected such that a cardioprotective effect during ischemia also results in diminished reperfusion-injury. Both IPC and RIC reduced mainly the second peak during normothermia, reflecting a predominant protection against reperfusion-injury. During mild hypothermia, however, only IPC had additive cardioprotective effect. The additive effect of mild hypothermia and IPC is consistent with findings by others^13–15^. Dote et al. showed that an increased IPC stimulus was needed during profound hypothermia of 25 °C^24^. In our study, we used a hypothermic target temperature of 34 °C because this was within the range used clinically for patients with cardiac arrest^25^ and acute myocardial infarction^26^. We found no need for a stronger IPC stimulus at 34 °C. LDH release patterns also suggest that the additional cardioprotective effect of IPC is associated with a modulation of reperfusion-injury. Herajarvi et al. demonstrated that RIC adds cardioprotective effect to hypothermia in a porcine model of hypothermic circulatory arrest that differed substantially from our model^27^. The effect of mild hypothermia in our rat hearts was powerful, challenging any demonstration of an additive effect of RIC.

We studied underlying mechanisms through metabolite release by microdialysis, glucose oxidation and phosphorylation of AKT and ERK. Animals subjected to mild hypothermia throughout the ischemic period (MH-Ischemia and MH-Total) had reduced interstitial lactate concentrations during ischemia and had smaller infarcts than normothermic controls. In the animals subjected to mild hypothermia during only the final 20 min of the ischemic period and during reperfusion, we found no reduction in lactate concentration despite a profound reduction in infarct size. Hence, lactate production measured by microdialysis may not be sufficiently sensitive to reflect cardioprotection. IPC, but not RIC, reduced the concentration of lactate during ischemia in normothermic animals. The lactate-reducing effect of IPC during ischemia may reflect an anti-ischemic component of IPC that is not mirrored by the LDH release. When combining IPC and mild hypothermia during ischemia, the concentration of lactate was low with no further significant reduction by IPC. In animals exposed to mild hypothermia during reperfusion, IPC significantly reduced interstitial lactate analogously to the normothermic groups. Considering the additive effect of IPC and mild hypothermia during ischemia, a further reduction in lactate does not seem to reflect the additional effect. The decrease in lactate concentrations during reperfusion was observed in all groups, with the exception of controls with hypothermia during ischemia only in the IPC experimental series. The result may reflect a shut down in metabolism, but we saw no similar effect in the corresponding IPC group.

Levels of succinate during ischemia and reperfusion have received significant interest since Chouchani et al. suggested that succinate is a universal metabolic signature of IR-injury^28^. The theory of reducing IR injury by inhibiting succinate accumulation during ischemia to moderate the reverse electron transport during early reperfusion has been discussed in recent studies. Andrienko et al. have queried the underlying mechanisms^29^. Our results show an increase in succinate during ischemia. However, the increase was not attenuated by either ischemic conditioning or mild hypothermia. Kohlhauer et al. investigated the combined effect of dimethyl malonate and mild hypothermia and demonstrated that mild hypothermia neither attenuated the succinate accumulation during ischemia nor modified the oxidation of succinate during reperfusion^30^. These findings are in accordance with our results. Pell et al. investigated the effect of IPC on levels of succinate, and demonstrated no impact of IPC on either accumulation during ischemia or metabolism during reperfusion^31^.

The effect of mild hypothermia on glucose oxidation during reperfusion differed depending on the timing of mild hypothermia in the protocol. Mild hypothermia during ischemia only increased glucose oxidation during reperfusion, whereas mild hypothermia in the other MH-protocols had no effect on glucose oxidation, even though some of the protocols were associated with infarct size reduction. IPC increased the oxidation of glucose during reperfusion in normothermic hearts confirming results by Støttrup et al.^32^. During mild hypothermic conditions IPC had no effect on glucose oxidation although the cardioprotective effect of IPC remained operative under these conditions. Our findings suggest that the cardioprotective effect of IPC and mild hypothermia are not crucially dependent on an effect on glucose oxidation. RIC did not affect glucose oxidation during reperfusion either during normothermia or hypothermia. The protective mechanism underlying RIC must be sought in other pathways.

Mild hypothermia may involve some of the same cardioprotective signalling pathways as ischemic conditioning, i.e. the reperfusion injury salvage (RISK) pathway and the survivor activating factor enhancement (SAFE) pathway^33^. In the present study we found that mild hypothermia activated the RISK pathway by increased phosphorylation of Akt to a similar level as IPC, but only when hypothermia was initiated during the last part of ischemia. None of the other hypothermic protocols increased phosphorylation of Akt, despite the same reduction in infarct size. Further, IPC did not activate phosphorylation of Akt during hypothermia. These differences in activation of Akt may suggest that activation of the RISK pathway is not prerequisite for the effect of IPC. Preserved ERK activity in the RISK pathway has been associated with mild hypothermia^34^, but in the present study we found no correlation between phosphorylated ERK, mild hypothermia, IPC, or RIC.

Our study has limitations. We used an isolated, non-working animal model of cardiac ischemia and reperfusion with glucose as the only substrate, which limits transferability to in-vivo physiology, because systemic responses to IPC, RIC, or mild hypothermia cannot be assessed in this model. Changes in metabolism and signalling pathways may be transient such that we may have missed the window of opportunity to detect every effect of the interventions. Mild hypothermia may induce hemodynamic and metabolic changes to a varying degree, depending on the timing of induction. We did not evaluate model dependent physiological responses to mild hypothermia in sham animals without ischemia and reperfusion and this should be considered a limitation. However, it does not detract from the validity of the differences that we observed between the study groups. We documented a cardioprotective effect using a conditioning protocol of three cycles of ischemia and reperfusion. We cannot rule out that a more intense RIC stimulus may have resulted in different outcome when added to mild hypothermia. Finally, we were forced to use two different rat strains and two underlying protocols. Rat strains are known to be affected differently by IR injury^35^, in this study illustrated by larger infarct size in Sprague Dawley (RIC series) rats compared to Wistar rats (IPC series), and we therefore chose to adjust the protocols to optimize the outcome and ensured a relevant control group for each series.

### Clinical perspective

We found a clear cardioprotective effect of mild hypothermia, and that the effect is crucially dependent on induction of cooling during ischemia—most likely well before reperfusion. While it may be achievable in elective surgical interventions, the premise may be difficult to obtain in unpredictable ischemia such as in acute MI patients. To achieve a sufficient cardioprotective effect to translate into a beneficial effect on mortality and morbidity in patients, a multi-target approach seems necessary and applicable in some settings. According to our results a combination of mild hypothermia and IPC may be attractive, but in combination with mild hypothermia, RIC may not be effective.

## Conclusion

In an isolated rat heart model, we found preserved and even additive cardioprotective effect of mild hypothermia and IPC, but not with RIC. The underlying mechanisms seem to differ between mild hypothermia and IPC, with mild hypothermia targeting the ischemic injury and IPC predominantly modulating reperfusion injury.

## Methods and materials

### Animals

As our supplier terminated the production of Wister rats, we used two different strains in our experiments. Male Wistar rats (300 g, M&B Taconic, Eiby, Denmark) were used for investigation of IPC, and male Sprague Dawley rats (300 g, M&B Taconic, Eiby, Denmark) were used for investigation of RIC. All animals were kept at a constant temperature of 23 °C with a 12-h light–dark cycle and allowed unlimited access to food and water. The study is in agreement with the Danish law for animal research and approved by the Danish Animal Experimental Inspectorate (Authorization No. 2012-15-2934-00623).

### Experimental protocols

Sensitivity to IR injury differed between our two rat strains. Accordingly, we chose different ischemia time in Sprague Dawley (30 min) and Wistar rats (40 min) as specified below in an attempt to equalize infarct size in our experiments. In addition, the differences between IPC and RIC approach required different preischemic handling.

In the IPC experimental series, the hearts from Wistar rats were isolated and subjected to Langendorff perfusion consisting of 40 min of pre-ischemic stabilisation, 40 min of global ischemia, and 120 min of reperfusion. IPC was induced before ischemia by 2 cycles of 5 min of global ischemia and 5 min of reperfusion^23^. Hypothermia was induced by perfusing the heart with Krebs–Henseleit buffer with a temperature of 34 °C instead of 37 °C and changing the temperature of the buffer surrounding the hearts to 34 °C^13,34^. Hypothermia was induced at different time points in the perfusion protocols: during ischemia only (MH-Ischemia), during the last 20 min of the ischemic period and throughout reperfusion (MH-Ischemia + Reperfusion), during reperfusion only (MH-Reperfusion), or through the total protocol (MH-Total). The hypothermia protocols were performed on control and IPC hearts.

In the RIC experimental series Sprague Dawley rats underwent either sham-procedure or RIC prior to isolation of the heart. RIC was induced using a tourniquet around the right hind leg, with three cycles of 5 min of ischemia and 5 min of reperfusion^36^. Ischemia was verified by paling of the foot, reperfusion was visualized by hyperaemia and a complete block of blood flow was verified by Doppler in selected animals. The hearts were then isolated and subjected to Langendorff perfusion consisting of 20 min of pre-ischemic stabilisation, 30 min of global ischemia and 120 min of reperfusion. Mild hypothermia was induced by the same method as in the IPC experimental series. In a clinical STEMI setting hypothermia can only be combined with RIC during ongoing ischemia, and therefore a protocol with hypothermia during the last half of the ischemic period and throughout reperfusion was used (MH-Ischemia + Reperfusion) with and without prior RIC (Fig. 1).

### Isolated perfused heart model

Rats from the IPC experimental series were anesthetized with a subcutaneous injection with 0.15 mL Dormicum (midazolam 5 mg/mL; Roche, Basel, Schwizerland) and 0.15 mL Hypnorm (fentanyl citrate 0,315 mg/mL, Fluanison 10 mg/mL: Janssen, Birkerød, Denmark). Tracheotomy was performed and the rats were connected to a rodent ventilator (Ugo Basile 7025 rodent ventilator, Comerio, Italy).

Rats from the RIC experimental series were anesthetized with pentobabiturate [65 mg/kg body weight (Skanderborg Pharmacy, Skanderborg, Denmark)]. Rats were then intubated and connected to a ventilator similar to that used in the IPC experimental series. The animals were ventilated with room air during the entire sham or RIC procedure, as well as during isolation of the heart.

Isolation of the heart was done according to standard procedure in our laboratory^37^. To isolate the heart a ligature with a tourniquet was placed around the aorta. The animals were heparinized by injection of 1,000 IU/kg heparin. Retrograde perfusion was established in situ with Krebs–Henseleit buffer [containing (in mmol/l) NaCl (118.5), KCl (4.7), NaHCO3 (25.0), glucosemonohydrate (11.1) MgSO4.7H2O (1.2), CaCl2 (2.4), and KH2PO4 (1.2)]. The hearts were rapidly excised and mounted in a Langendorff apparatus and perfused at a constant pressure of 80 mmHg. The perfusion buffer was oxygenated with 95% O_2_ and 5% CO_2_ to maintain a pH of 7.35–7.45. The temperature was kept constant at 37 ± 0.5 °C during normothermia and 34 ± 0.5 °C during hypothermia. To induce rapid changes in myocardial temperature, we switched both the perfusion buffer and the buffer in the organ bath surrounding the heart. This allowed us to change target temperature within approximately 30 s. Intramyocardial temperature was kept at the same levels and monitored with a temperature probe placed in the free wall of right ventricle (Harvard Apparatus, Natick, MA). A balloon-catheter (size 7, Hugo Sachs Electronics, March-Hugstetten, Germany) connected to a pressure transducer, was inserted into the left ventricular cavity, for continuous hemodynamic measurements. The balloon volume was adjusted to obtain a left ventricular end-diastolic pressure of 7–10 mmHg. The coronary flow was continuously measured by an in-line flow probe (Hugo Sachs Electronics, March-Hugstetten, Germany). All data was acquired and digitally analysed using a dedicated core software platform (Notocord Hem evolution, Croissy sur Seine, France).

Exclusion criteria were LVDP below 110 mmHg at the end of stabilisation, coronary flow of more than 20 mL/min, failure to reach target temperature according to protocol, or continuous ventricular fibrillation during stabilisation or reperfusion.

### Infarct size

At the end of reperfusion hearts were immediately frozen at − 80 °C and subsequently cut into ≈ 1.5 mm slices according to standard procedure^38^. Slices were immersed in 1% 2,3,5-triphenyltetrazolium chloride (Sigma, St. Louis, Mo, USA) at 37 °C. Hearts were stored in 4% formaldehyde (Lillies Solution, VWR—Bie & Berntsen, Herlev, Denmark) for 20–28 h to enhance the contrast between vital and infarcted tissue. Each heart slice was weighed and scanned on a flatbed scanner (Epson Perfection V600 Photo Scanner, Epson America Inc.). The area of left ventricle (LV), which corresponds to the AAR, and area of infarction were assessed manually by observer delineation using computer assisted planimetry (ImageJ 1.46r, Wayne Rasband, National Institutes of Health, USA). Infarct size was expressed as a percentage infarct size of AAR. Measurements were weighted with the weight of each individual slice. All analyses were performed in a blinded manner.

### Biochemical markers of myocardial ischemia (temporal LDH release)

Effluent samples for measuring LDH release were collected throughout the protocol. The samples were immediately cooled on ice and stored at − 80 °C until analysis. LDH content in the effluent was measured using a LDH activity kit (K726-500, BioVision Inc., Milpitas, CA, USA) according to the manufacturer’s instructions. The measured concentrations were corrected for coronary flow and heart weight and expressed as mU × g^−1^ × min^−1^.

### Microdialysis

Myocardial microdialysis was performed to assess interstitial concentrations of citric acid cycle intermediates and glycolytic end products. A microdialysis probe (membrane length 4 mm cut-off 6 kDa;AgnTho’s AB, Sweden) was inserted into the free wall of the left ventricular in the isolated heart enabling sampling with a perfusion rate of 1 μL/min over 10 min with deoxygenated Krebs–Henseleit buffer. Perfusion rate was controlled by using a Univenter 801 syringe pump (AgnTho’s AB, Sweden). TCA metabolites in microdialysis samples were quantified by ultraperformance liquid chromatography and tandem mass spectrometry (Waters UPLC and Xevo TQ-S mass spectrometer, Waters Corp., Manchester, UK), as described in detail elsewhere^39^. Results were corrected for previously determined relative recovery rates (lactate: 37%, succinate: 26%)^39^.

### Tracer

Rates of glucose oxidation were measured using statically tritium labelled glucose isotopes (D-[6-^3^H]-glucose)^40^. Pre-experimental buffer samples were drawn to assess baseline specific activity per μmol glucose in the buffer. Glucose oxidation was quantified by ^3^H_2_O production by oxidation of D-[6-^3^H]-glucose in the citric acid cycle. To determine the production of ^3^H_2_O during the protocol, buffer samples of coronary effluent were collected at several timepoints. The specific activity was analysed by separation of labelled glucose from ^3^H_2_O by anion exchange chromatography on AG 1-X8 resin columns (Bio-Rad, Hercules; CA; USA) according to the manufacturer’s instructions. The purified ^3^H_2_O was suspended in 10 mL Ultima Gold scintillation solution (Perkin-Elmer, Shelton, CT, USA) and quantified by beta-scintillation on a TriCarb 2900TR liquid scintillation analyser (Packard, Perkin, IL, USA) in detection per minute (dpm). Rates of glucose utilization were corrected for heart weight and coronary flow.

### Intracellular signalling pathways (Western blotting)

In separate series of animals, the hearts were freeze clamped and left ventricular biopsies were collected after 10 min of reperfusion and stored at − 80 °C until use. Left ventricular biopsies were processed and western blot was performed as previously described^41^.

Western blot was performed with primary antibodies against phosphorylated and non-phosphorylated Akt and Erk (Akt (pan) (C67E7) Rabbit mAb #4691, Phospho-Akt (Ser473) (D9E) XP Rabbit mAb #4060, p44/42 MAPK (Erk1/2) (137F5) Rabbit mAb #4695, Phospho-p44/42 MAPK (Erk1/2) (Thr202/Tyr204) (D13.14.4E) XP Rabbit mAb #4370.) The primary antibodies used in this study were all purchased from Cell Signaling Technology (Danvers, MA, USA).

The membrane was analyzed using the ChemiDoc MP Imaging System (Bio-Rad, Hercules, CA, USA). Western blots were normalized to total protein measured by the Stain-Free technology^42^.

### Statistical analysis

Data are presented as mean ± SEM, unless otherwise indicated. Data were compared using ANOVA with a post hoc test when appropriate (Sidak's multiple comparisons test) and ANOVA with repeated measurements (or equivalent non-parametric test). All statistical calculations were performed using GraphPad Prism (GraphPad Software, CA, USA). P < 0.05 was considered significant. The required sample size was estimated from previously published work using the isolated heart model^37^.

## Supplementary Information

Supplementary Information.
