# Supplementary material for: Cardioprotective effect of combination therapy by mild hypothermia and local or remote ischemic preconditioning in isolated rat hearts
Source: Sci Rep. 2021 Jan 11;11:265. doi: 10.1038/s41598-020-79449-x (PMC7801421; doi:10.1038/s41598-020-79449-x)
Supplement: Supplementary file 1 — Supplementary Information. [file 41598_2020_79449_MOESM1_ESM.docx]

**Supplementary Information**

**Cardioprotective effect of combination therapy by mild hypothermia and local or remote ischemic preconditioning in isolated rat hearts**

Marie V. Hjortbak*, MD^1^, Nichlas R. Jespersen, MD, PhD^1^, Rebekka V. Jensen, MD, PhD^1^, Thomas R. Lassen, MD^1^, Johanne Hjort, MD^1^, Jonas A. Povlsen, MD, PhD^1^, Nicolaj B. Støttrup, MD, PhD^1^, Jakob Hansen, PhD^2^, Derek J. Hausenloy, MD, PhD^3,4,5,6,7^, Hans Erik Bøtker, MD, PhD, DMSc^1^

**Supplementary results**


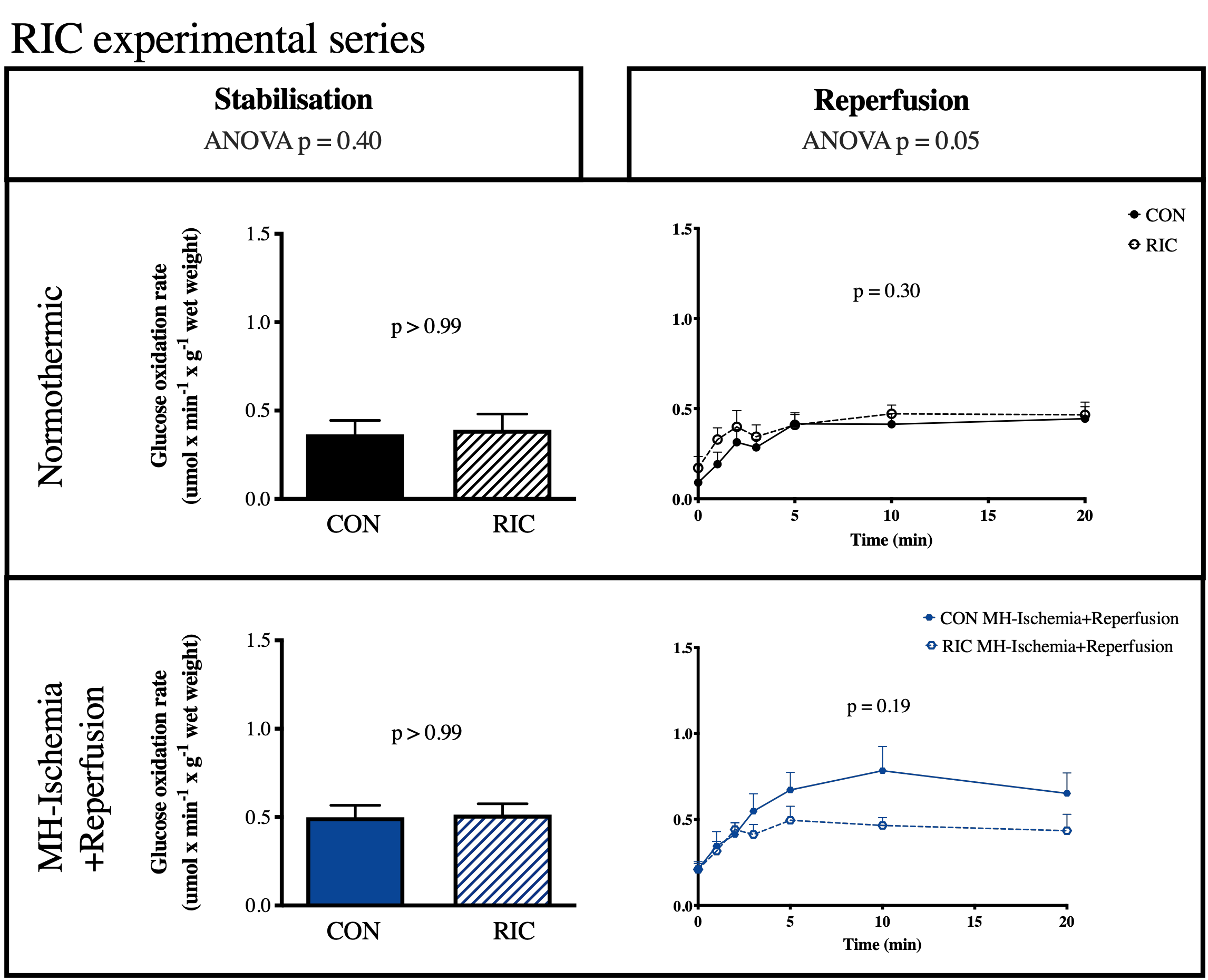


**Figure S1. Glucose oxidation.**

CON: control, IPC: ischemic preconditioning, MH-Ischemia: mild hypothermia during ischemia, MH-Reperfusion: mild hypothermia during reperfusion, MH-Total: mild hypothermia through the total protocol.


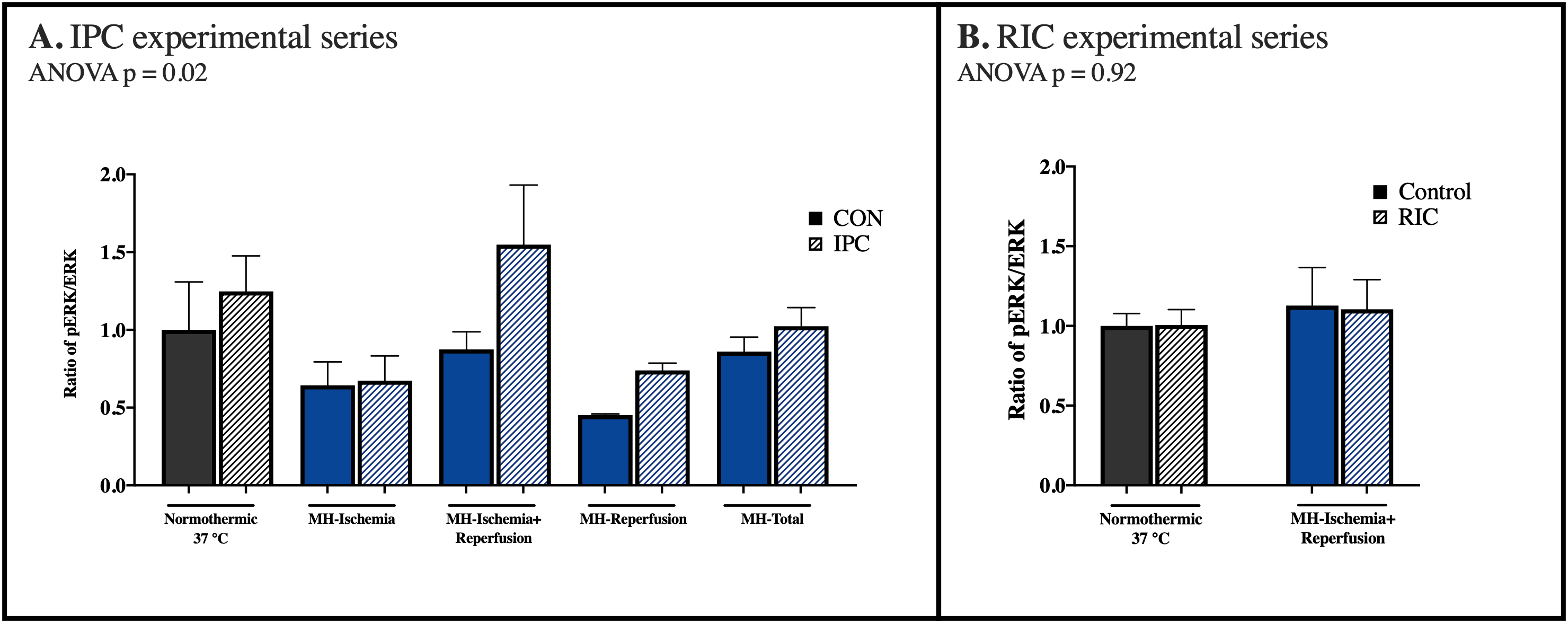


**Figure S2. Phosphorylation of ERK.**

**(**A) IPC experimental series, (B) RIC experimental series. CON: control, IPC: ischemic preconditioning, RIC: remote ischemic preconditioning. MH-Ischemia: mild hypothermia during ischemia, MH-Ischemia+Reperfusion: mild hypothermia during half of the ischemia and throughout reperfusion, MH-Reperfusion: mild hypothermia during reperfusion, MH-Total: mild hypothermia through the total protocol.

Data is presented as a ratio with normothermic CON as reference group.

**Technical limitations**

Technical malfunction caused loss of data on glucose oxidation in hearts from MH-Ischemia+Reperfusion in the IPC experimental series. Normothermic control-hearts were insufficiently loaded with glucose-tracer prior to baseline sampling, so data represented in the CON group rely on combined data from CON MH-Ischemia and CON MH-Reperfusion, which are transferrable at the baseline sampling point. Microdialysis yields small sample size. In MH-Total group, we experienced technical malfunction that resulted in very small samples sizes and a reduced number of samples, which did not allow us to perform statistically robust analyses.

**Full length uncropped western blot gels**


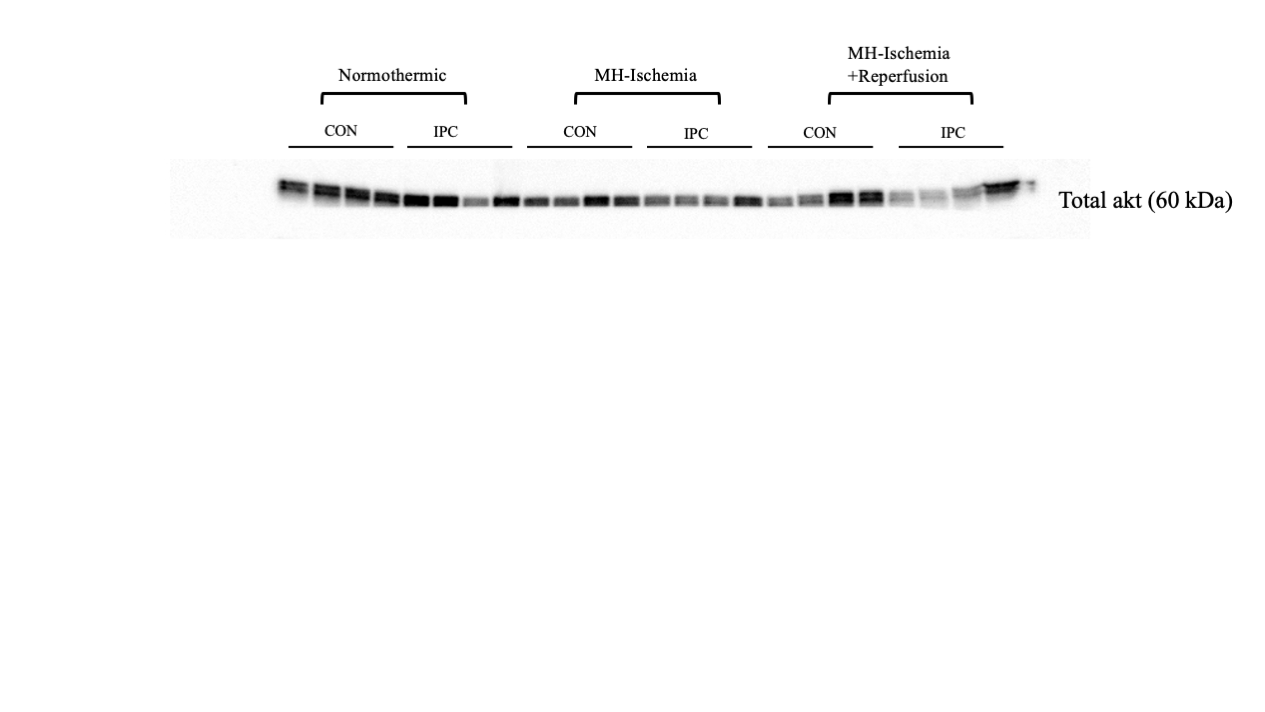
a.

**
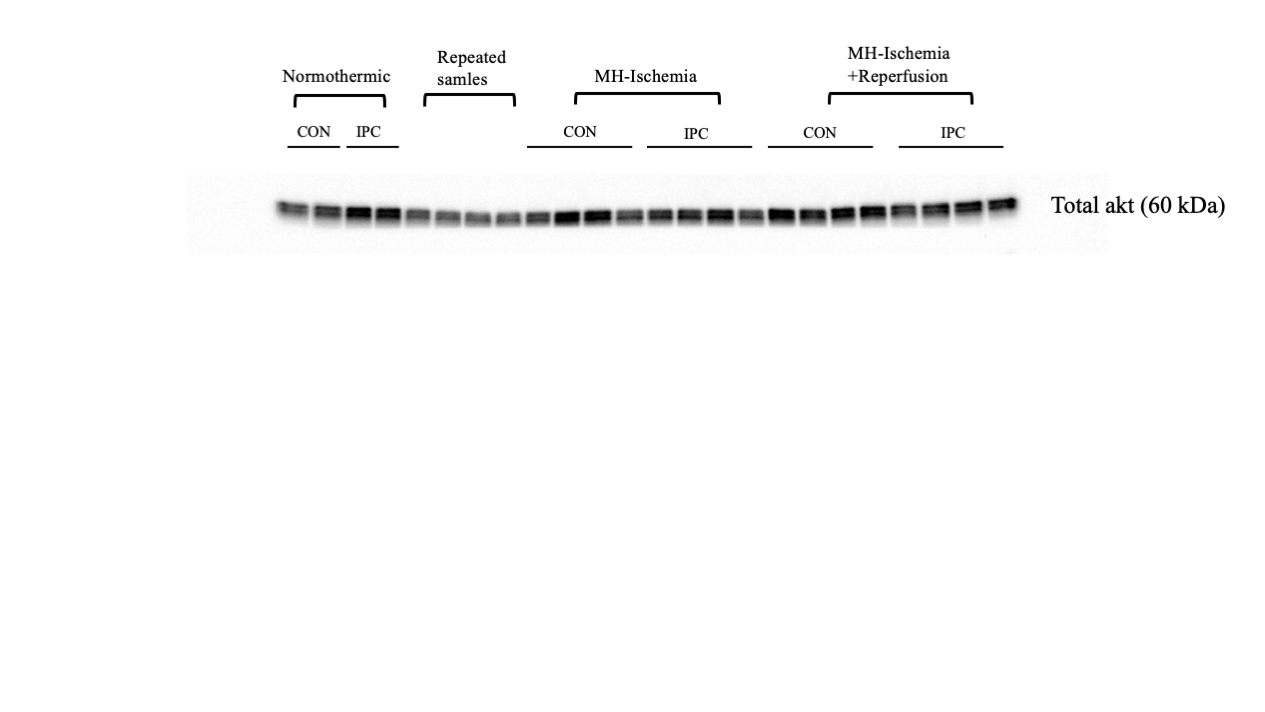
**b.

**Figure S3. Full length blot of total Akt.** Uncropped images used for expression of total Akt in figure 6c. The first gel in figure 6c is shown in (a), and the second gel is shown in (b). On the gel shown in b, the first four samples are repeated samples from the normothermic control and IPC groups, used to secure continuous results and normalise the data (only CON). n=4 in all groups.


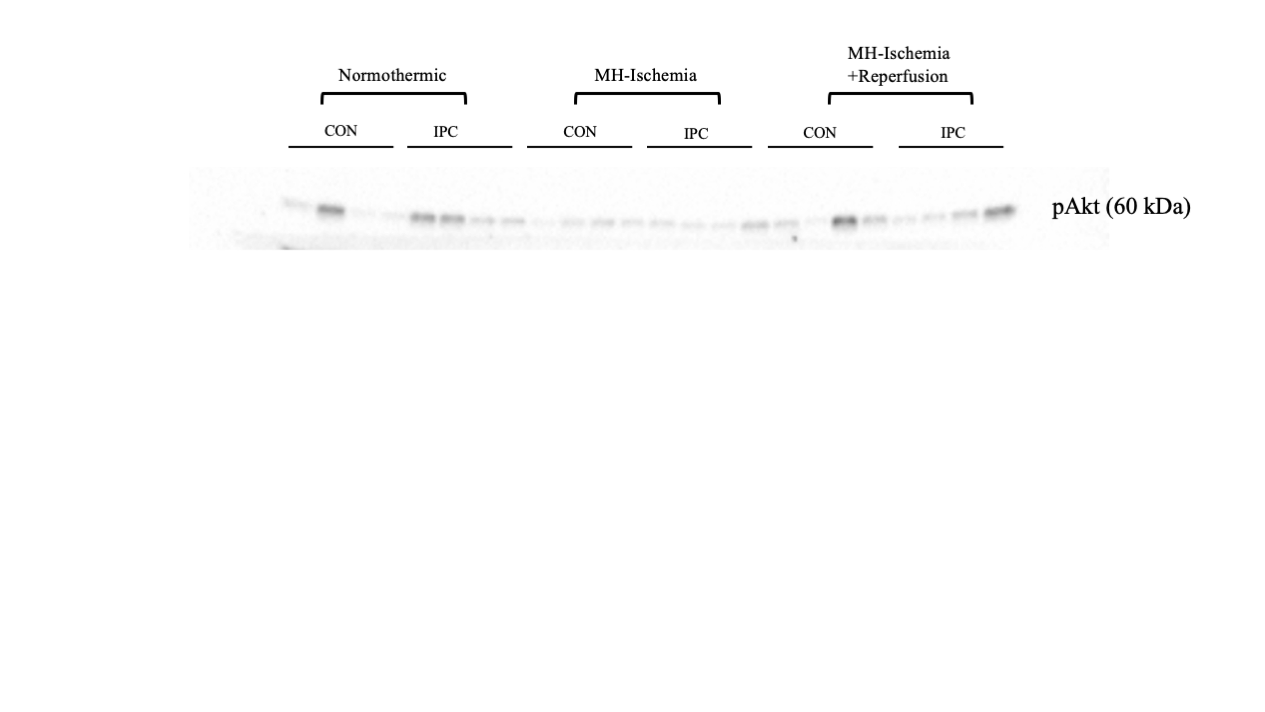
a.


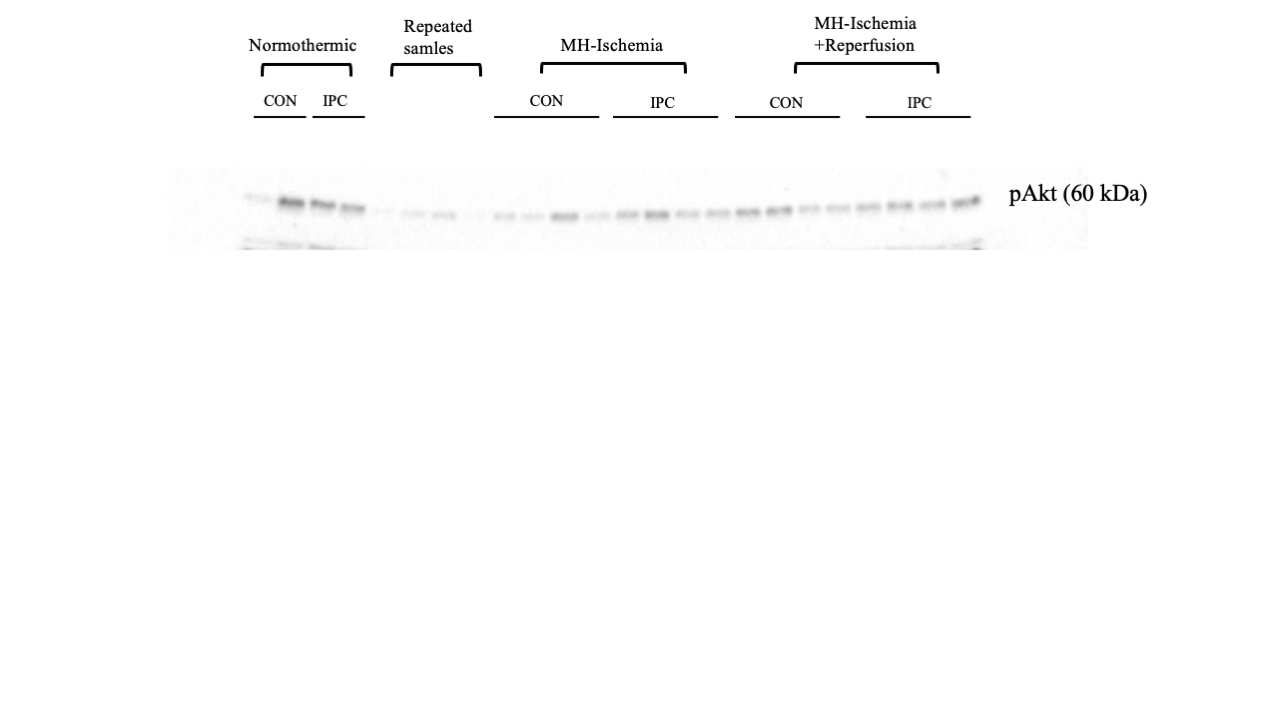
b.

**Figure S4. Full length blot of phosphorylated Akt.** Uncropped images used for expression of phosphorylated Akt in figure 6c. The first gel in figure 6c is shown in (a), and the second gel is shown in (b). On the gel shown in b, the first four samples are repeated samples from the normothermic control and IPC groups, used to secure continuous results and normalise the data (only CON). n=4 in all groups.


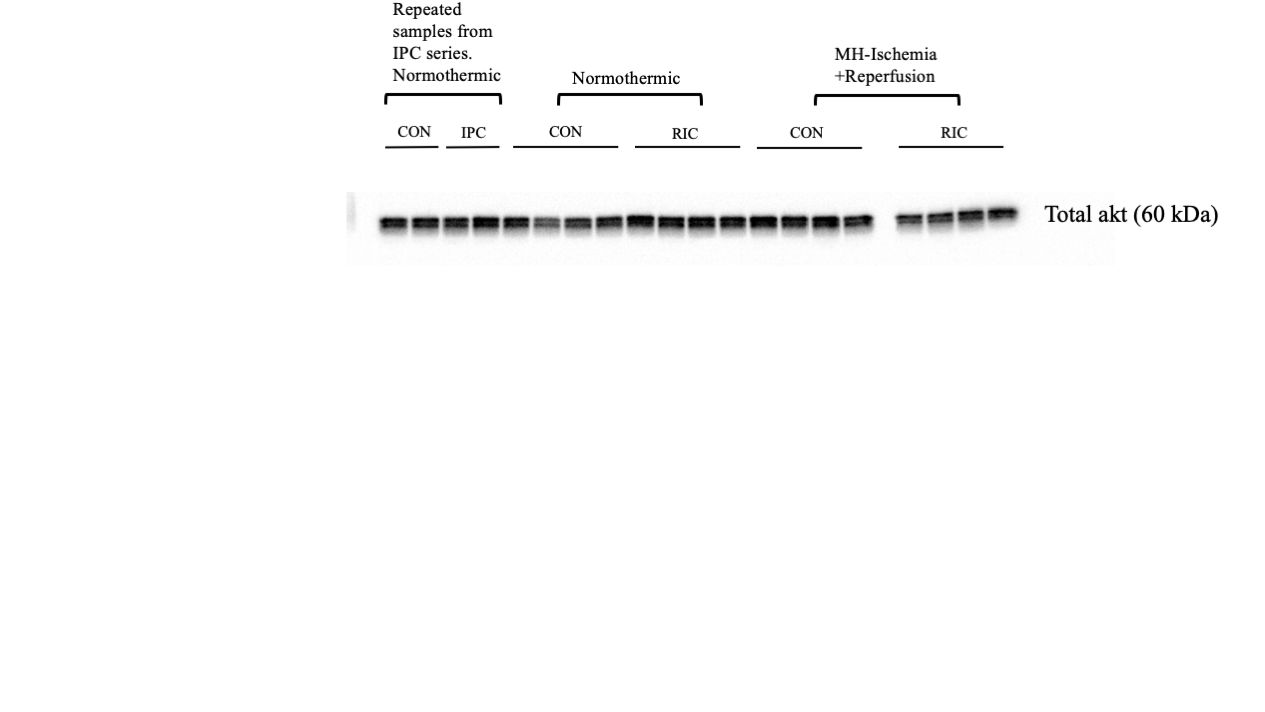


**Figure S5. Full length blot of total Akt for RIC experimental series.** Uncropped images used for expression of total Akt in figure 6d. The first four samples are repeated samples from the IPC experimental series: normothermic control and IPC groups, used to secure continuous results and normalise the data (only CON). n=4.


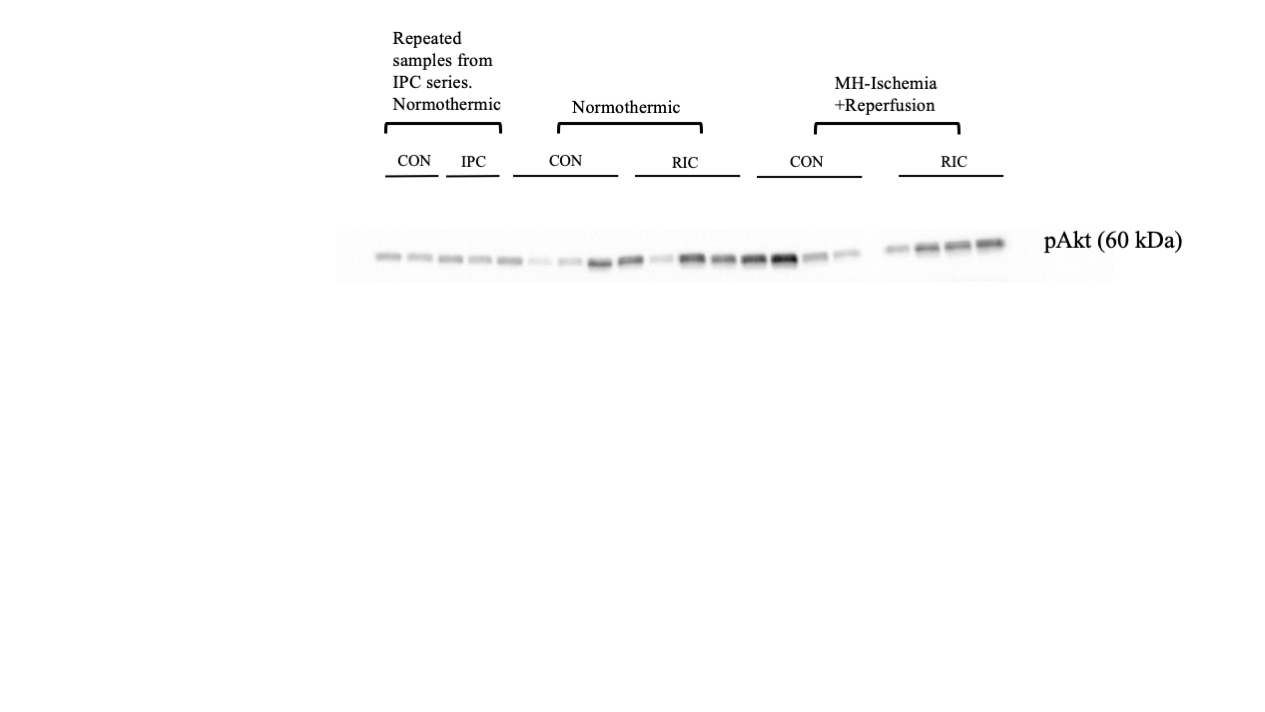


**Figure S6. Full length blot of phosphorylated Akt for RIC experimental series.** Uncropped images used for expression of phosphorylated Akt in figure 6d. The first four samples are repeated samples from the IPC experimental series: normothermic control and IPC groups, used to secure continuous results and normalise the data (only CON). n=4.


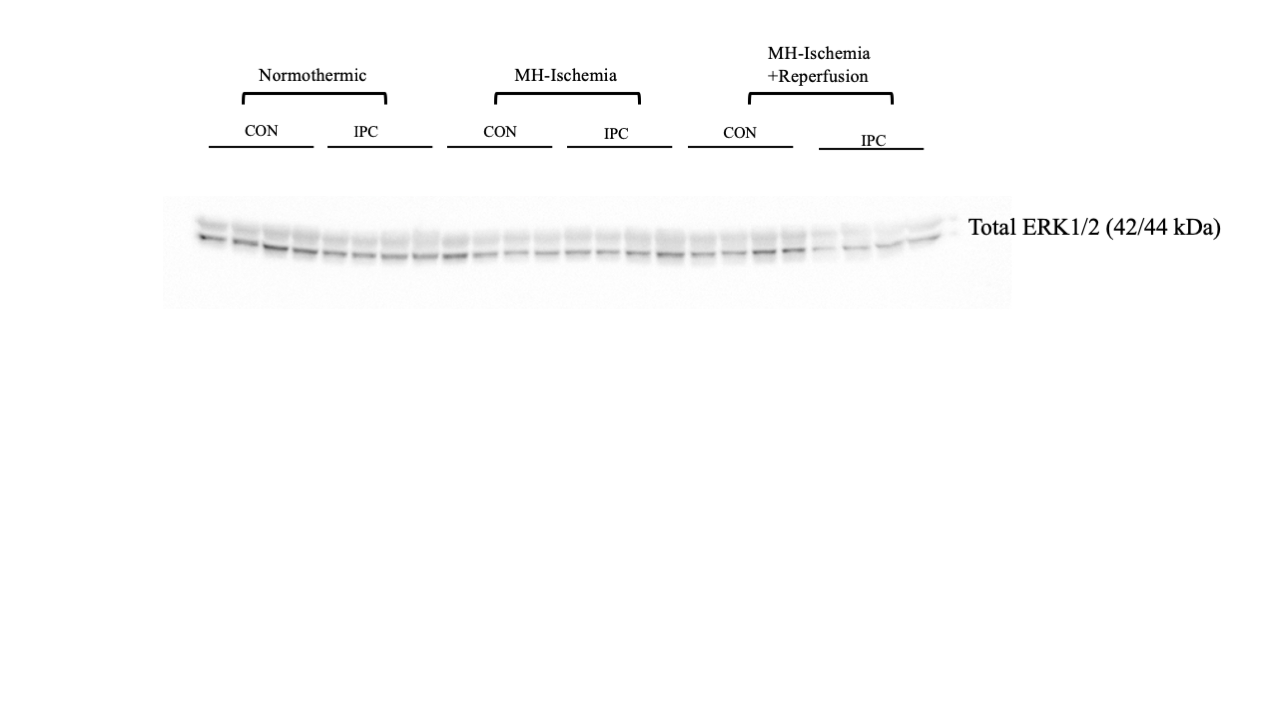
a.

**
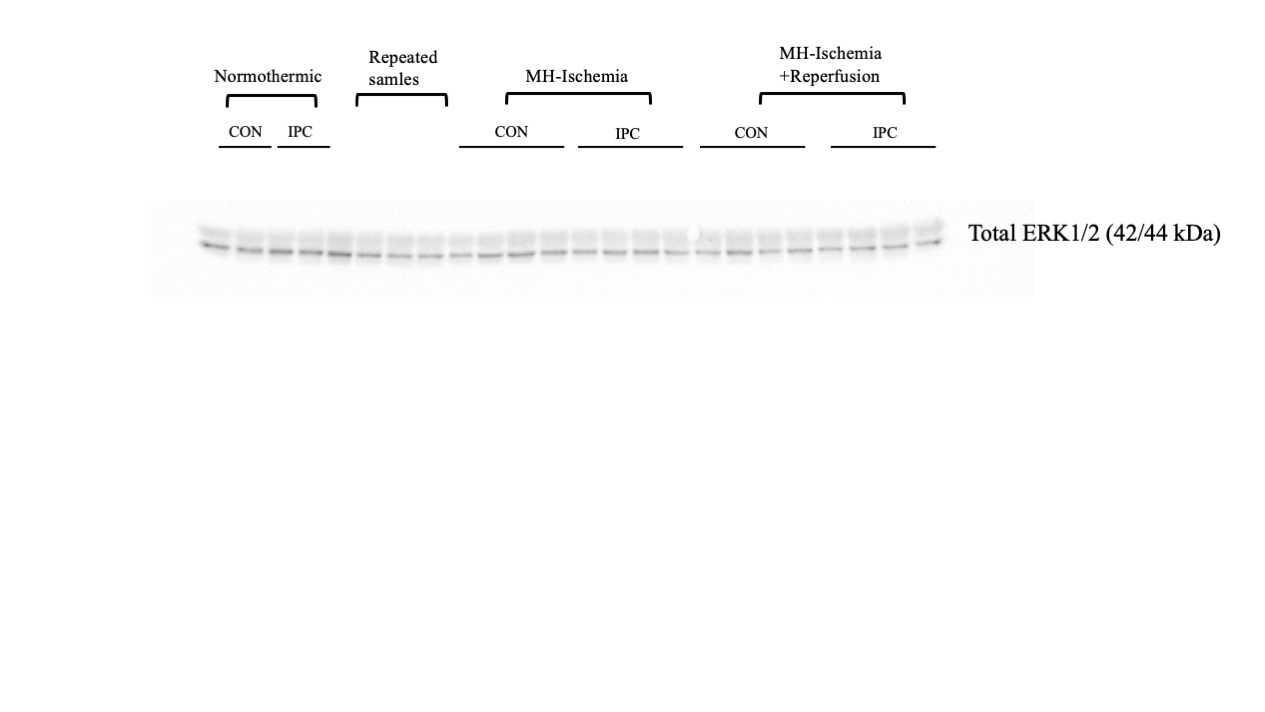
**

b.

**Figure S7. Full length of blot of total ERK1/2.** Uncropped images used for expression of total ERK1/2 in figure S2. The samples are organised similarly to the Akt experiments. n=4 in all groups.


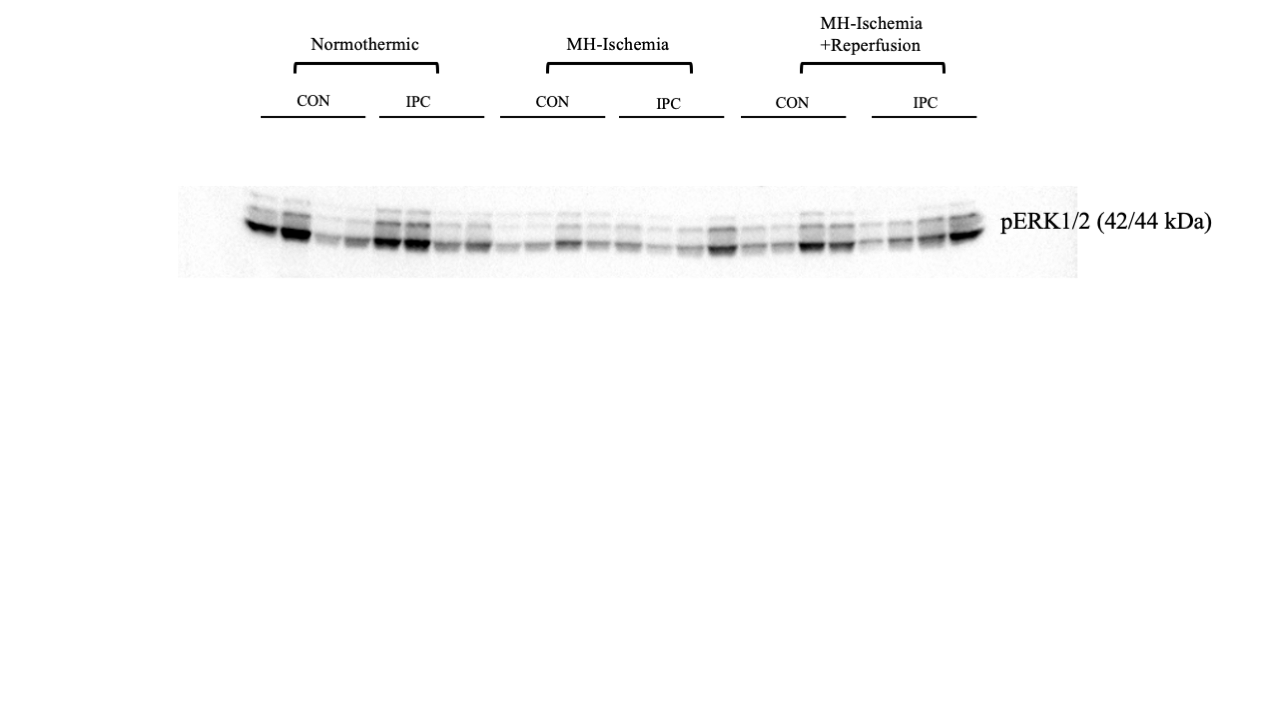
a.


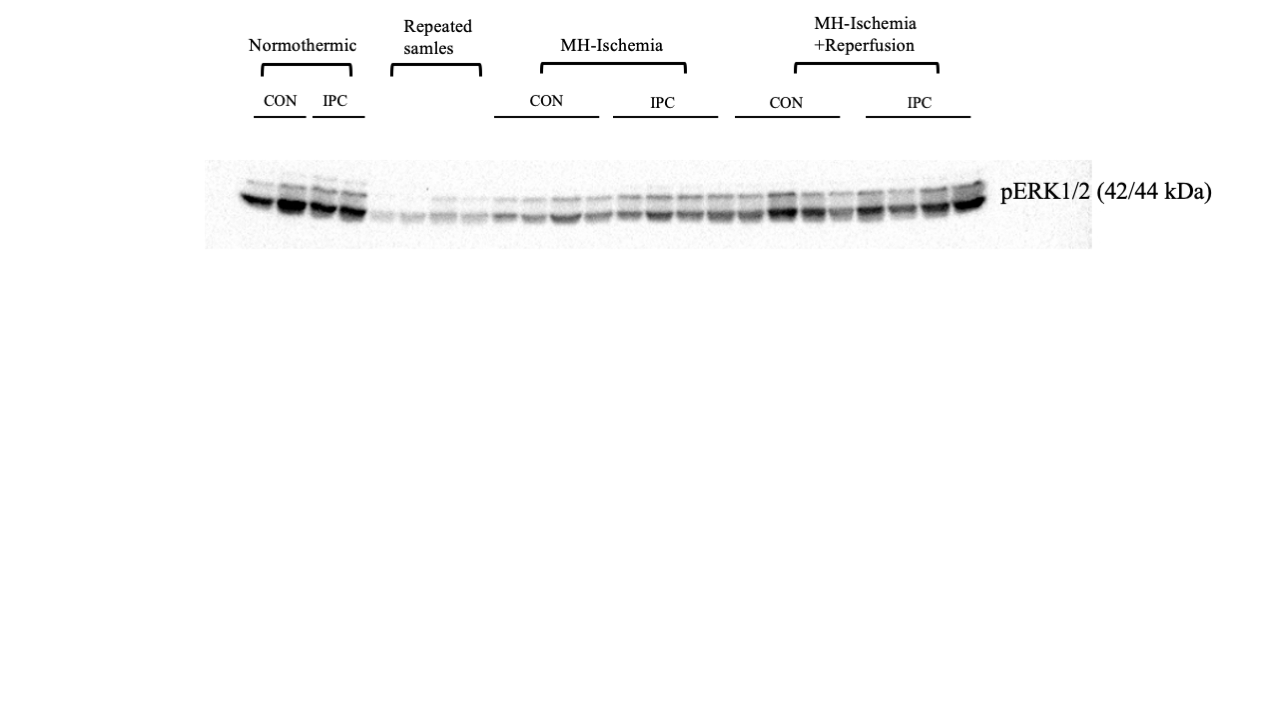
b.

**Figure S8. Full length of blot of phosphorylated ERK1/2.** Uncropped images used for expression of phosphorylated ERK1/2 in figure S2. The samples are organised similarly to the Akt experiments. n=4 in all groups.


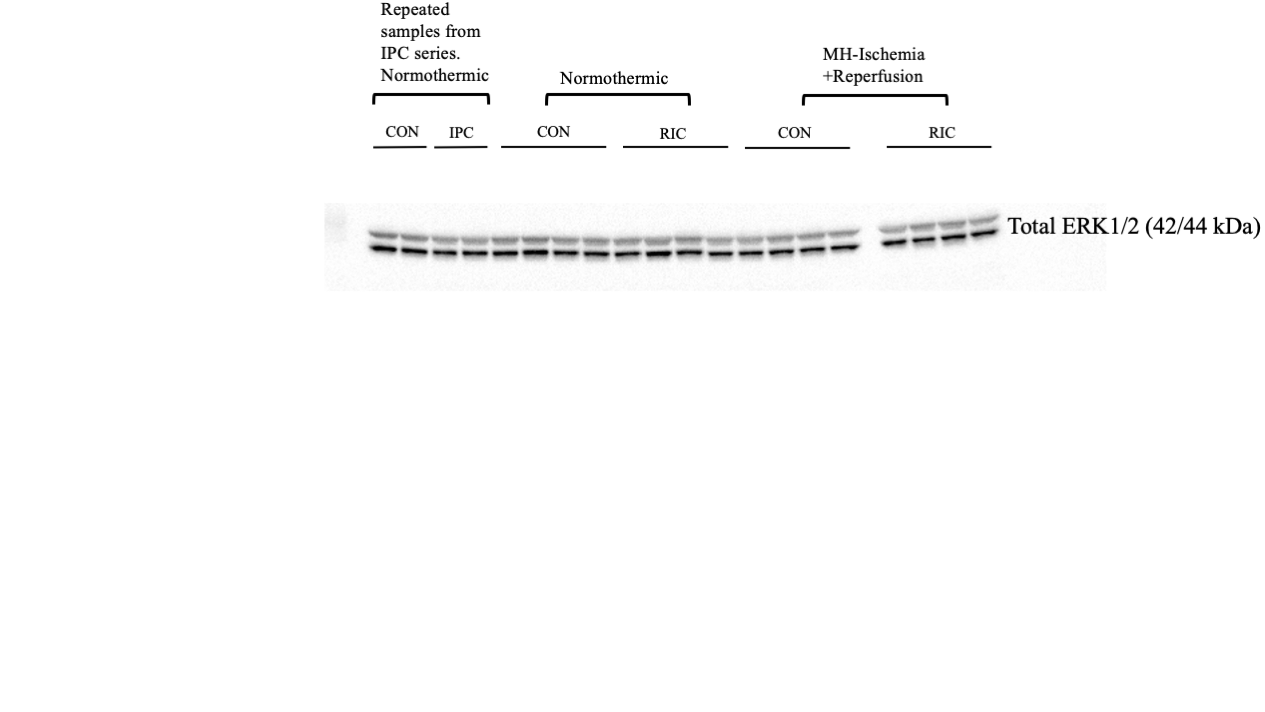


**Figure S9. Full length blot of total ERK for RIC experimental series.** Uncropped images used for expression of total ERK in figure S2. The first four samples are repeated samples from the IPC experimental series: normothermic control and IPC groups, used to secure continuous results and normalise the data (only CON). n=4.

**
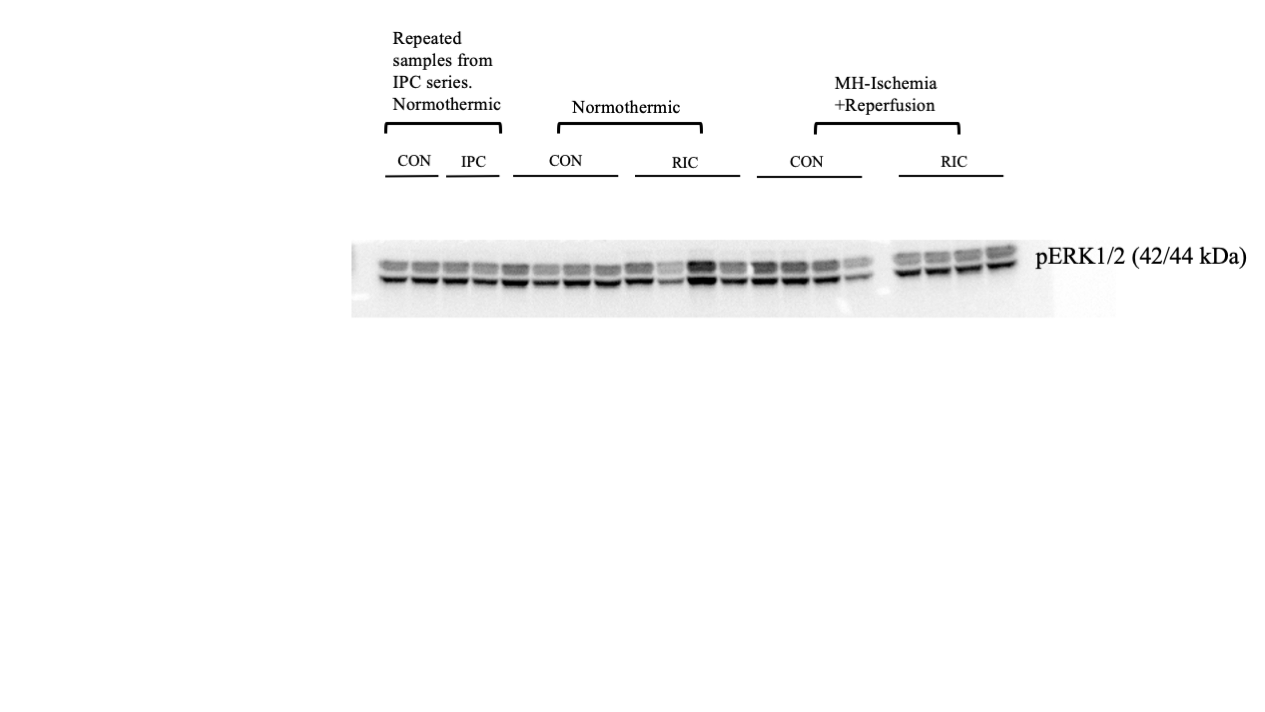
**

**Figure S10. Full length blot of phosphorylated ERK for RIC experimental series.** Uncropped images used for expression of phosphorylated ERK in figure S2. The first four samples are repeated samples from the IPC experimental series: normothermic control and IPC groups, used to secure continuous results and normalise the data (only CON). n=4.
